# Comparative genomics and evolution of the HSP90 family of genes across all kingdoms of organisms

**DOI:** 10.1186/1471-2164-7-156

**Published:** 2006-06-17

**Authors:** Bin Chen, Daibin Zhong, Antónia Monteiro

**Affiliations:** 1 School of Life Sciences, Southwest University, Chongqing 400715, P.R. China; 2 Department of Biological Sciences, The State University of New York at Buffalo, NY 14260, USA

## Abstract

**Background:**

HSP90 proteins are essential molecular chaperones involved in signal transduction, cell cycle control, stress management, and folding, degradation, and transport of proteins. HSP90 proteins have been found in a variety of organisms suggesting that they are ancient and conserved. In this study we investigate the nuclear genomes of 32 species across all kingdoms of organisms, and all sequences available in GenBank, and address the diversity, evolution, gene structure, conservation and nomenclature of the HSP90 family of genes across all organisms.

**Results:**

Twelve new genes and a new type HSP90C2 were identified. The chromosomal location, exon splicing, and prediction of whether they are functional copies were documented, as well as the amino acid length and molecular mass of their polypeptides. The conserved regions across all protein sequences, and signature sequences in each subfamily were determined, and a standardized nomenclature system for this gene family is presented. The proeukaryote HSP90 homologue, HTPG, exists in most Bacteria species but not in Archaea, and it evolved into three lineages (Groups A, B and C) via two gene duplication events. None of the organellar-localized HSP90s were derived from endosymbionts of early eukaryotes. Mitochondrial TRAP and endoplasmic reticulum HSP90B separately originated from the ancestors of HTPG Group A in Firmicutes-like organisms very early in the formation of the eukaryotic cell. TRAP is monophyletic and present in all Animalia and some Protista species, while HSP90B is paraphyletic and present in all eukaryotes with the exception of some Fungi species, which appear to have lost it. Both HSP90C (chloroplast HSP90C1 and location-undetermined SP90C2) and cytosolic HSP90A are monophyletic, and originated from HSP90B by independent gene duplications. HSP90C exists only in Plantae, and was duplicated into HSP90C1 and HSP90C2 isoforms in higher plants. HSP90A occurs across all eukaryotes, and duplicated into HSP90AA and HSP90AB in vertebrates. Diplomonadida was identified as the most basal organism in the eukaryote lineage.

**Conclusion:**

The present study presents the first comparative genomic study and evolutionary analysis of the HSP90 family of genes across all kingdoms of organisms. HSP90 family members underwent multiple duplications and also subsequent losses during their evolution. This study established an overall framework of information for the family of genes, which may facilitate and stimulate the study of this gene family across all organisms.

## Background

HSP90 proteins, named according to the 90 kDa average molecular mass of their members, are highly conserved molecular chaperones that account for 1–2% of all cellular proteins in most cells under non-stress conditions [1]. They participate in the regulation of the stress response [2,3] and, when associated with other co-chaperones, function in correctly folding newly synthesized proteins, stabilizing and refolding denatured proteins after stress, preventing misfolding and aggregation of unfolded or partially folded proteins, and assisting in protein transport across the endoplasmic reticulum (ER) and organellar membranes [4-8]. HSP90 members have key roles in the maturation of signal transduction proteins, like hormone receptors, various kinases, nitric oxide synthase and calcineurin [9-13]. Through these substrates, they regulate diverse cellular processes. The combination of these functions appears to result in a role as a capacitor of phenotypic variation: decreasing cytosolic HSP90 function in *Drosophila melanogaster *and *Arabidopsis thaliana *results in the appearance of phenotypes that depend on the genetic background but which would normally be cryptic [7,14].

HSP90 expression is also associated with many types of tumors including breast cancer, pancreatic carcinoma, human leukemia, systemic lupus erythematosus, as well as multidrug resistance [1]. HSP90 inhibition provides a recently developed, important pharmacological platform for anticancer therapy [15].

Most Eubacteria were thought to have a single homologue of HSP90 known as HTPG (high temperature protein G), located on the chromosomal genome, whereas Archaebacteria lack a HSP90 representative [16,17]. In eukaryotes all HSP90 genes are located on the nuclear genome, but their proteins function in the cytosol, ER, chloroplast and mitochondria [1,18,19]. The cytosolic members, called HSP90 (90 kDa heat-shock protein) in the *sensu stricto *appear in two major isoforms, Hsp90-α (inducible form) and Hsp90-β (constitutive form) [1,20,21]. These two isoforms are the result of a gene duplication about 500 million years ago [22]. The ER member, generally called Grp94 (94-kDa glucose-regulated protein), was thought to exist in all eukaryotes except fungi, which appear to have lost it, and was suggested to have originated via gene duplication very early in eukaryote evolution [23]. A chloroplast homologue that is most similar in sequence to ER Grp94 has been found in three plant species [17,24]. The mitochondrial homologue, TRAP (tumor necrosis factor receptor-associated protein), is most closely related to Eubacterial HTPG sequences, which suggests it originated from a HTPG-like ancestor [17,25]. As a distinctive feature, TRAP possesses a unique LxCxE motif that is absent in all other HSP90 family members, and depends on stress kinases for its transcriptional activation [26].

There have been several studies that attempt to draw the phylogenetic relationships across all HSP90 family members [17,23,24,27]. However, due to the limited number of sequences used, and the lack of genome-wide information, the diversity of HSP90 family members and their evolutionary relationships are still not completely resolved.

The evolution of protein-coding genes is frequently applied to infer patterns of organismal evolution, but gene trees are sometimes not consistent with species trees. One problem that can be encountered, especially when using genes that belong to a large gene family, is the use of paralogous instead of orthologous gene copies in phylogenetic reconstruction. Using information from complete genomes, however, and including all known family members minimizes this problem. Because the Hsp90 family of proteins exists in all organisms except Archaea [23], and because they are quite conserved, they provide an excellent model for understanding the evolutionary pattern of the majority of life on our planet. In the present study we analyzed 32 complete genomes, across all kingdoms of life, and retrieved all HSP90 family of sequences available within these genomes. In addition, we investigated the complete genomes of 18 and 99 further species of Archaea and Bacteria, respectively. We aimed to: 1) determine the diversity and distribution of HSP90 family members throughout all organisms, 2) document the chromosomal location and exon splicing patterns of HSP90 genes, and the amino acid (a.a.) length and molecular mass of HSP90 proteins, and predict whether these genes are functional, 3) identify the conserved regions and the signature sequences of each class of HSP90 family members, 4) analyze the evolution of the gene family members throughout all kingdoms of organisms, 5) provide a case-study of the use of these proteins for addressing the phylogenetic relationship of all organisms, and 6) propose a standardized nomenclature system for the members of this gene family based on our earlier work [28]. This study establishes an overall framework of information for the family of HSP90 genes, which may facilitate and stimulate the study of this gene family across all organisms.

## Results

### Diversity and nomenclature of the HSP90 family of genes

There are a total of 103 genes belonging to the HSP90 family on the 32 genomes analyzed (Table 1, Table 2). Out of these 103 genes, 87 are putative functional genes and 16 are pseudogenes, based on functional motif/domain analysis (see the following section). Twelve genes are newly predicted in the study, and 2 protein sequences are newly deduced from mRNA sequences available in databases. The new sequences are in Additional File 1. The number of HSP90 family members is highest on vertebrate genomes, with a total of 4–16 genes, followed by Plantae with 3–8 genes, invertebrates (including "invertebrate" Deuterostomes, Ecdysozoans and Lophotrochozoans) with 3–5 genes, Protista with 2–4 genes, and Bacteria with only 0–2 genes (Table 1). In Archaea, only *Methanosarcina mazei *has a HSP90-like gene, and in viruses, there is no HSP90 homologue.

Due to the limit of sequences that PSI-BLAST database searching can report, the 6 query sequences used to probe the databases (see Methods) each retrieved 1000 similar sequences with the statistical significance threshold of 0.005. Removal of redundant sequences from the collection of 6000 sequences resulted in 1015 protein sequences that came from different submissions. From these, 452 complete sequences were confirmed to belong to the family, through similarity comparison after sequence alignment, and subsequently used for the analysis of protein features. From these complete sequences, 197 representative sequences, distributed across 146 species, and 128 genera in Archaea, Bacteria, Protista, Plantae, Fungi and Animalia, were selected for phylogenetic analysis. We established three-letter abbreviations of their species names (Table 3) that were used throughout this study.

We divided the gene family into five subfamilies named HSP90A, HSP90B, HSP90C, TRAP and HTPG. Our earlier study proposed a new nomenclature system for the HSP90 family of genes on the human genome [28], which was approved by the HUGO Gene Nomenclature Committee [29]. The system uses the root names HSP90A, HSP90B, and TRAP to indicate cytosolic, ER, and mitochondrial HSP90 homologues, respectively. HSP90A was further divided into two classes: HSP90AA for conventional Hsp90-alpha and HSP90AB for Hsp90-beta. Following Schroda [30] we used HSP90C to name the homologues found in the chloroplast. The present study retains the nomenclature system above (which is strongly supported in our analysis below) and also retains the conventional name for the bacterial homologue of HSP90, HTPG. Following the guidelines for human and mouse gene nomenclature [31,32], we used a number following the root/subfamily names to encode the gene in the subfamily, and a "P" at the end of the gene name to indicate a possible pseudogene (Table 2). We used the three-letter code for different species (Table 3) to distinguish the species of origin for homologous genes. The codes for species are put in parenthesis and prefixed to the gene names (see Table 2). In our search for an outgroup to root the HSP90 gene family we found that the Archaean *Methanosarcina mazei *HSP90-like gene, (MMA)NP_634445.1 (Table 2), can be aligned with HTPG members despite not sharing the conserved residues/motifs present throughout all HSP90 members, and being significantly longer (982 a.a.) than bacterial HTPGs (588–681 a.a.). When blasting this sequence against the protein database in GenBank, we found that its closest relatives exist in bacteria (e.g. *Rhodopseudomonas palustris*, ZP_00811094.1, 26% identity; *Frankia *sp., ZP_00568340.1, 26% identity). We decided to use this HSP90-like protein and one of its bacterial relatives as outgroups to HTPG members.

The single-exon *HTPG *genes are present in a variable number of copies across the 109 complete bacterial genomes investigated. Five species have two copies of *HTPG*, 79 species have a single copy, and 25 species have no homologous *HTPG *genes. Presence or absence of *HTPG *on a particular genome is not strongly correlated with membership in any one bacterial group. For instance, whereas in Cyanobacteria and Spirochaeteles, *HTPG *exists in all species investigated, and in Lactobacillales and Mollicutes no *HTPG *was identified in any of the species investigated, in the genera *Corynebacterium*, *Bacillus *and *Haemophilus, HTPG *was only found in some species but not in others.

**Figure 1:**
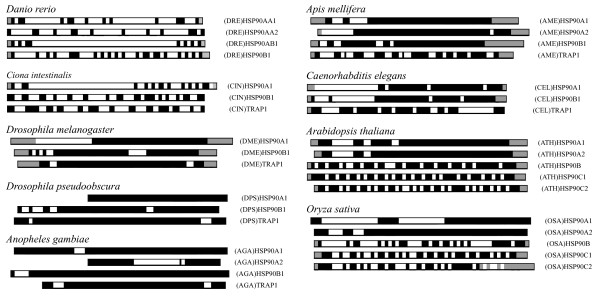
Exon splicing patterns of representative genes of the HSP90 family, identified with the genomic study and drawn to scale relative to the number of nucleotides present in each region. The black-filled rectangles depict protein-coding sequences, gray-filled rectangles represent untranslated regions, and unfilled rectangles represent introns.

TRAP genes have 3–19 exons and are present only in Animalia. HSP90C genes have 10–21 exons and only exist in Plantae. HSP90B genes have 1–18 exons and exist in all species of Protista, Plantae and Animalia, and some species in Fungi. HSP90A genes have 1–12 exons and exist in all eukaryotic kingdoms. HSP90A were divided into two subdivisions, HSP90AA and HSP90AB (Table 1) in Vertebrates, and HSP90C was divided into HSP90C1 and HSP90C2 in Plantae. The exon splicing patterns of representative members of the HSP90 family are shown in Figure 1.

### Protein features and conserved regions

A total of 456 complete amino acid sequences (452 available in databases and 4 newly predicted) can be reliably aligned, and the alignment of representative sequences from each of the 5 subfamilies is shown in Figure 1. Based on the overall alignment, we divided the protein sequence into 3 conserved regions (I, II and III) and four variable regions (A, B, C and D). In a previous study, Obermann and others [33] divided HSP90 protein sequences into five domains: the N-terminal domain [(HSA)HSP90AA1 residues 1–236], Charged domain 1 (237–271), Middle domain (272–617), Charged domain 2 (618–628) and C-terminal domain (629–732). In order to characterize the difference between subfamilies based on the overall alignment, we made further subdivisions to the domains mentioned above. We divided the N-terminal domain into the Variable regions A [(HSA)HSP90AA1 residues 1–17] and Conserved region I (18–224), identified a new Conserved region II (290–617) for the Middle domain, and a new Variable region C (618–628) for the Charged domain 2, and divided the C-terminal domain into the Conserved region III (629–699) and Variable regions D (670–732)(Fig 1). We also replaced the Charged domain 1 (237–271) by the extended Variable region B (225–289) in order to reflect the sequence variation found in our alignment for this area.

**Figure 2:**
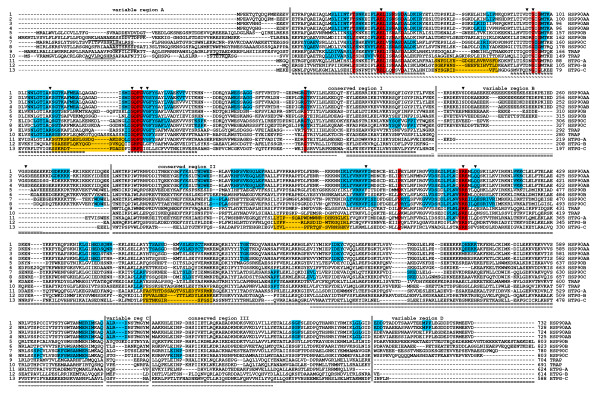
Alignment of representative amino acid sequences of the HSP90 family of proteins, showing their conserved/variable regions, functionally important residues, and functional domains. Amino acid residues completely conserved throughout the HSP90 family are shaded in red, and those conserved throughout each subfamily are in green. The regions that distinguish HTPG Groups A, B and C are shaded in yellow. Gaps are marked with"-", the last residue in each line is assigned a number and subfamily names are indicated at the end of each line. The conserved/variable regions are separated by "||" with the names above the alignment, and the cleavage sites located just before the underlined residues. "▼" stands for the functionally important residues experimentally identified: E47 (refers to HSP90AA1) for ATP hydrolysis; D93 for ATP binding [33]; G95, G132, G135, G137, and G183 for both GA and p23 binding; K112 for GA binding [72]; R400 and Q404 for ATPase activity; F369 for interdomain interaction [63]; S231 and S263 for phosphorylation by casein kinase II [74]. The conserved and functional domains are indicated by: "≈" for HSP90 protein family signature; "=" for HSP90 protein; "~" for histidine kinase-like ATPases; "∞" for four-helical cytokine; and "..." for Glutamic acid-rich region. The number of each sequence represents: 1. (HSA)HSP90AA1, 2. (GGA)HSP90AA1, 3. (HSA)HSP90AB1, 4. (GGA)HSP90AB1, 5. (HSA)HSP90B1, 6. (ATH)HSP90B1, 7. (ATH)HSP90C1, 8. (ATH)HSP90C2, 9. (HSA)TRAP1, 10. (DME)TRAP1, 11. (ECO)HTPG1, 12. (BFR)HTPG1.

The residues that are conserved across all members of a subfamily or across all HSP90 family members, and that function as signature sequences, are summarized below (see also Figure 2 and Table 4). HTPG genes, like most bacterial genes, have only one exon. Their proteins are 588–681 a.a. long, have no signal peptide, and lack the Variable regions A, B and D (Figure 2). This subfamily of proteins are the shortest in the HSP90 family, with molecular mass 66.7–78.0 kDa (Table 1), and are also the most variable with no recognizable signature sequences.

TRAP, HSP90C and HSP90B proteins possess a signal peptide that allows their export into mitochondria, chloroplasts, and ER, respectively [1,19] (Figure 1). TRAP genes have 3–19 exons, their proteins lack the Variable regions B and D, and their mature protein sequences are 644–687 a.a. long with a molecular mass of 74.8–77.9 kDa (Figure 1, Table 1). Five conserved motifs can each distinguish TRAP members from other subfamily members (Table 4). HSP90C genes have 10–21 exons. Their proteins have a shorter Variable region B relative to HSP90A and HSP90B, are 756–785 a.a. long, and have a molecular mass of 84.2–89.0 kDa in their mature status. Eight unique motifs can distinguish HSP90C members from other subfamily members (Table 4). HSP90B genes have 1–18 exons. Their proteins have a shorter Variable region B relative to HSP90A, and are 695–800 a.a. long with a molecular mass of 79.5–91.5 kDa in mature status. We cannot find a completely conserved motif that alone distinguishes HSP90B proteins from the others but the simultaneous presence of three conserved motifs, as a group, can separate these proteins from other family members. A fourth motif, the most-C-terminal motif KDEL of HSP90B, previously used as the signature motif for members of this subfamily, and thought to be the ER retention signal, was here found to be not completely conserved across all HSP90B subfamily members. With the exception of the L residue, which is conserved across all sequences examined, at the K site, 38.7% of the sequences in our aligment had an H, A, E, R, S or N residue instead. At the D site, G or N appeared in several cases [e.g. (TCR)HSP90B1, (SMA)AAF66929.1], and at the E site, this residue was replaced by a D in a few cases [e.g. (LML)XP_847734.1]. HSP90A genes have 1–12 exons. Their proteins have a very short Variable region A but a full-length Variable region B, and they are 689–854 a.a. long with molecular mass of 78.3–98.1 kDa in mature status. Eleven conserved motifs can each distinguish HSP90A proteins from other subfamily members (Table 4). The MEEVD motif at the C-terminal end was considered functional and characteristic of cytosolic HSP90 [23]. This motif is quite conserved but there are exceptions. In several cases the second E can be a Q or a K [e.g. (TCR)HSP90A1, (DDI)HSP90A1], and its D is absent in (CIN)HSP90A1. HSP90AB sequences can be easily distinguished from HSP90AA sequences by the former having a unique LKID motif, and the latter having 7 unique motifs (Table 4, Figure 1). There are, however, a small number of intermediate type sequences that fall between the typical HSP90AA and HSP90AB (Figure 4).

Seventeen a.a. residues are completely conserved throughout all sequences (Table 4). These residues as well as various conserved/functional domains shared by all HSP90 members may be fulfilling HSP90-specific functions. InterProScan and ScanProsite searches explored such domains (Figure 1). The "HSP90 protein" sequence [residues 196–732 in (HSA)HSP90AA1, document # PF00183 of InterPro database] exists in all HSP90 sequences. There are several smaller "signature" sequences (residues 18–61, 88–123, 131–153 and 182–218, InterPro doc PR00775), an ATP-binding domain (residues 40–193, ATPases, InterPro doc PF02518 and SM00387), a Glutamic acid-rich motif (residues 223–268, PROSITE doc PS50313), and a four-helical cytokine region (residues 518–668, InterPro doc SSF47266) (Figure 1). Other domains/motifs that occur in some of these HSP90 a.a. sequences include a Lysine-rich domain (PROSITE doc PS50318), a Protein kinase C phosphorylation site (PROSITE doc PS0005), a Casein kinase II phosphorylation site (PROSITE doc PS0006), a N-glycosylation site (PROSITE doc PS0001), a Tyrosine kinase phosphorylation site (PROSITE doc PS0007), a Tyrosine sulfation site (PROSITE doc PS0003), a cAMP- and cGMP-dependent protein kinase phosphorylation site (PROSITE doc PS0004), a N-myristoylation site (PROSITE doc PS0008), a Bipartite nuclear targeting site (PROSITE doc PS00015), a Leucine zipper domain (PROSITE doc PS00029), and a Amidation site (PROSITE doc PS00009).

### Phylogenetic relationships

Out of the 197 sequences selected for the phylogenetic analysis, 63 belong to HTPG, 15 to TRAP, 6 to HSP90C, 31 to HSP90B, and 78 to HSP90A. In order to maximize the number of characters that are alignable within and across closely related subfamily members, we divided these sequences into 2 subsets, HTPG+TRAP, and HSP90A+B+C, and deleted the smaller non-alignable regions in each of these alignments. A total of 595 of 624 a.a [refer to (ECO)HTPG1] in the HTPG+TRAP alignment and 679 of 732 a.a. [refer to (HSA)HSP90AA1] in the HSP90A+B+C alignment were finally used in the analysis. The HSP90-like (MMA)NP_634445.1 and (RPA)ZP_00811094.1 were used as a combined outgroup for the HTPG+TRAP alignment, whereas (SCO)HTPG1 and (BSU)HTPG1 were used as an outgroup for the HSP90A+B+C alignment.

**Figure 3:**
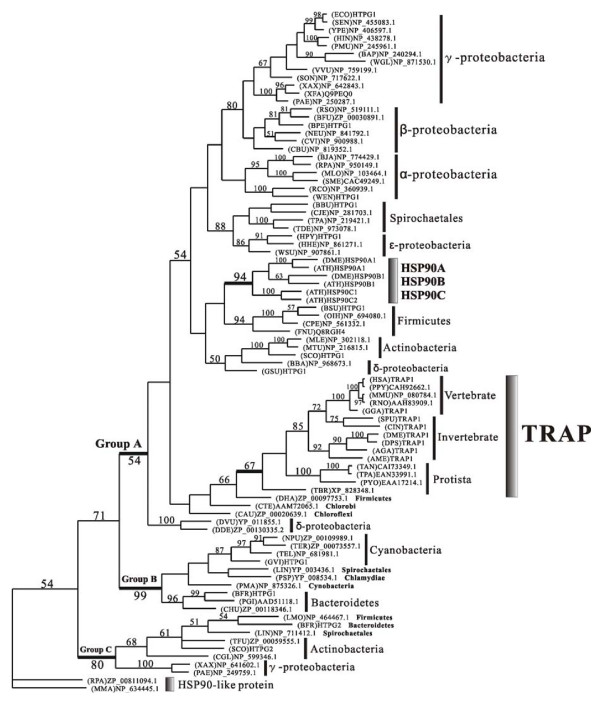
Most parsimonious tree inferred from HTPG and TRAP proteins with representative HSP90A, HSP90B and HSP90C members, rooted with HSP90-like proteins existing in Archaea and Bacteria. Bootstrap percentages of 5000 replicates are shown above the branches where they exceed 50%. Branch lengths are proportional to the number of character changes.

**Figure 4:**
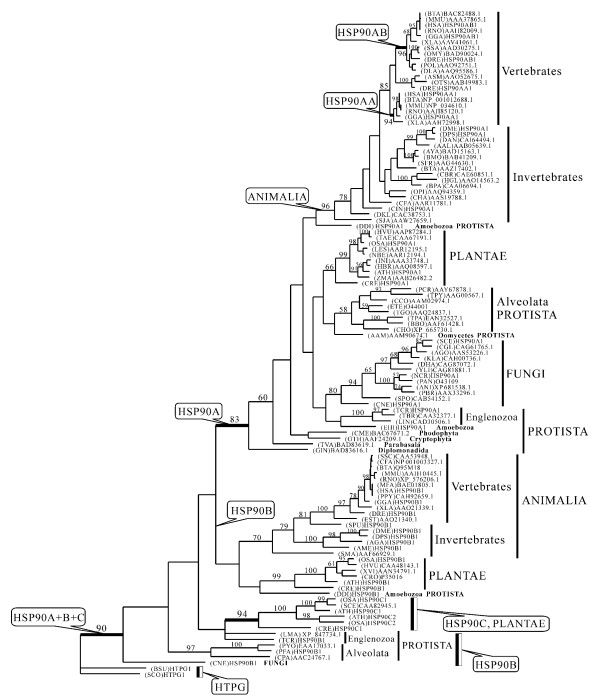
Most parsimonious tree inferred from HSP90A+B+C proteins, rooted with bacteria HTPG proteins. Bootstrap percentages of 5000 replicates are shown above the branches where they exceed 50%. Branch lengths are proportional to number of character changes.

The analysis of HTPG+TRAP data led to a single most parsimonious tree (Figure 3), with tree length = 15337, consistency index (CI) = 0.362, homoplasy index (HI) = 0.638, retention index (RI) = 0.527, and rescaled consistency index (RC) = 0.190. Eighty-four of the total 601 a.a. characters were constant, 117 were parsimony-uninformative and 584 were parsimony- informative. Based on the tree topology, we divided the protein sequences into three main groups, Groups A, B, and C. Sequence length differences in four different positions along the alignment can differentiate the members of these three groups (Figure 1). Our Groups B and C were strongly supported with 99% and 80% bootstrap values, respectively, whereas Group A was only moderately supported with a 54% bootstrap value. Group B+C was supported with a 71% bootstrap value. Groups B and C comprise only HTPG proteins, whereas Group A includes most HTPG, and all HSP90A, HSP90B, HSP90C and TRAP proteins. In Group A, HSP90A+B+C was a strongly supported monophyly with a 94% bootstrap value, and with Firmicutes HTPG proteins being the clade's closest relatives. TRAP proteins are monophyletic, supported with a 67% bootstrap value and also have Firmicutes HTPG proteins as their closest relative. Spirochaetales HSP90 proteins are found in all three groups, those from γ-proteobacteria, Firmicutes and Actinobacteria are found in Groups A and C, and those from Bacteroidetes are found in Groups B and C. The most parsimonious tree (10282 steps, CI = 0.372, HI = 0.628, RI = 0.680, RC = 0.253) for the HSP90A+B+C alignment is shown in Figure 4. Out of 681 total characters, 38 are constant, 93 are parsimony-uninformative and 605 are parsimony-informative. The nodes for HSP90A, HSP90C and ingroup (HSP90A+B+C) are all strongly supported with bootstrap values of 83%, 94% and 90%, respectively. In vertebrates HSP90A has duplicated into HSP90AA and HSP90AB, both clades supported with high bootstrap values (96% and 94%), but there are some intermediate sequences that according to their signature sequences should belong to HSP90AA but that in this analysis fall at the base of the HSP90AB clade. The HSP90A monophyletic clades that evolved within Animalia, Plantae, and Fungi are all well supported by bootstrap values of 96%, 66% and 80%, respectively, and at the base of these clades are HSP90A subfamily members belonging to the Protista. (GIN)BAD83616.1 of Diplomonadida is at the root of the Eukaryote members. HSP90B is not a monophyletic clade, and it originated earlier relative to HSP90A. HSP90C originated from HSP90B, and duplicated into HSP90C1 and HSP90C2 in higher plants with 100% and 98% bootstrap support, respectively. (CNE)HSP90B1 of Fungi did not cluster with the Animalia sequences, as expected. Instead it appeared as the most basal sequence within the HSP90A+B+C ingroup, but without >50% bootstrap support.

## Discussion

### Diversity, distinctive features and nomenclature of HSP90 family of genes

The present study presents the first comparative genomic study and evolutionary analysis of the HSP90 family of genes across all kingdoms of organisms. Based on the phylogeny of their proteins and the cell compartments the proteins function in, we divided the gene family into 5 subfamilies, HSP90A, HSP90B, HSP90C, TRAP, and HTPG, and established a new nomenclature system. This system may serve as a model for the nomenclature of other chaperone gene families (e.g. HSP100, HSP70, HSP60, and small HSP). HSP90A and HSP90B exist in all eukaryotic kingdoms, whereas HTPG, TRAP and HSP90C occur only in Bacteria, Animalia, and Plantae, respectively. HSP90A duplicated into HSP90AA and HSP90AB in vertebrates, and HSP90C duplicated into HSP90C1 and HSP90C2 in higher plants.

Seventeen completely conserved a.a. residues serve as markers to recognize any HSP90 family member, whereas membership into each subfamily is assigned by additional specific signature motifs, with the exception of HTPG subfamily members.

From the 32 complete genomes investigated we found that the number of exons in HSP90 genes range from 1 to 21, the mature a.a. length from 588 to 854 residues, and the molecular mass of mature proteins from 66.7 to 98.1 kDa. The HSP90 family of genes (with the exception of HTPG, and some HSP90A and HSP90B genes) contain a large number of introns. These data contradict earlier work [34] that suggested that HSP genes generally lacked introns, which would facilitate their rapid expression while avoiding incorrect RNA spicing due to heat stress. Other HSP families of genes have not been systematically studied, and possibly that some genes in these families also contain introns.

### HTPG subfamily of genes and the origin of HSP90

**Figure 5:**
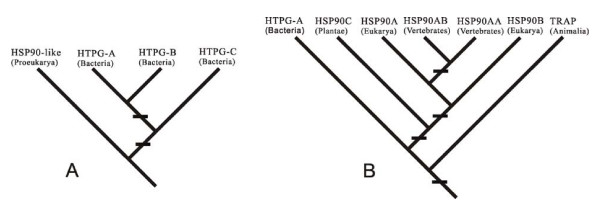
Schematic diagram representing the evolution of proeukaryotic (A) and eukaryotic (B) HSP90 family of genes. The filled bars indicate gene duplication events. A) We propose that HSP90-like genes in Bacteria and Archaea are basal to HTPG genes that only exist in Bacteria. HTPG genes underwent two gene duplication events that gave rise to three groups (Group A, B and C). B) Eukaryotic members were derived from HTPG Group A, and evolved into four subfamilies TRAP (mitochondrial), HSP90A (cytosolic), HSP90B (ER) and HSP90C (chloroplast) throughout three gene duplication events. One additional gene duplication event led to the division of HSP90A into HSP90AA and HSP90AB in vertebrates. The organismal groups in parenthesis indicate where these HSP90 gene copies are found.

The alignment and phylogeny of HTPG proteins divided them into three groups, each containing unique signature sequence and high or moderate bootstrap support. Importantly, our genomic study showed that at least 5 species of Bacteria (e.g. *Streptomyces coelicolor *and *Bacteroides fragilis*) each have two HTPG gene copies, each copy belonging to different groups. Each HTPG group also contains representatives of Spirochaetales, γ-proteobacteria, Firmicutes, Actinobacteria and Bacteroidetes. Unlike the proteins in other subfamilies, HTPG proteins are so divergent that they do not have a common signature sequence. Our data suggest that HTPG underwent two separate gene duplication events during its evolution (Fig 5A). Because the evolutionary origin and diversification of proeukaryotes is not yet resolved with certainty [35-37], this limits our ability to discuss the evolution of HTPG. Stechman and Cavalier-Smith [17] rooted their tree using a single and incomplete Ecobacteria (*Chloroflexus aurantiacus*) HTPG copy and did not consider HTPG gene duplication events. As a result, they placed TRAP close to the base of all HSP90 family members. We decided to root the HSP90 tree based on an ancient gene duplication event. Our genome searches identified HSP90-like proteins that exist in Archaea and Bacteria. They are most similar to HTPG proteins but do not have the conserved marker residues characteristic of all HSP90 family members. Using these HSP90-like sequences at the root of the tree, we postulate a gene duplication event that produced Group C sequences at the base of the HSP90 family tree, and a sequence that gave rise, via another duplication event to Groups A and B (Fig 5A). This scenario is supported by the presence of 4 variable regions in HTPG proteins (Fig 1), where the sequence length in each of these regions increases from Group C to Groups A and B. In additional support for this hypothesis is the observation that Group A sequences and all eukaryote homologues have 2 common signature sequences. This hypothesis needs to be further addressed when more genome sequences, especially those from basal bacteria, become available.

In order to identify the type of organism where HSP90 proteins originated we observe that not all bacterial groups (e.g. Lactobacillales and Mollicutes) have HTPG genes on their genomes, and neither does Archaea. If we assume that Archaea and gram-positive bacteria are the most ancient lineages within prokaryotes [35], then HSP90 proteins could have arisen within a Bacterial lineage that later gave rise to the eukaryotic cell. If we assume, however, that Archaea is a derived lineage from gram-positive bacteria [36,37], then HTPG proteins originated before the origin of Archaea, but were subsequently lost in this lineage. It will be possible to elucidate these two alternatives when the origin of the tree of life is better understood. Also, present-day bacteria taxonomy [38] is not completely consistent with our tree topology for HTPG proteins. HSP90 genes might provide a handle to elucidate bacterial evolution and taxonomy in future work.

### TRAP subfamily of genes

Consistent with Emelyanov [24] and Stechmann and Cavalier-Smith [17], our data suggests that TRAP evolved from within the ancestral eukaryotes, and was not derived from an endosymbiont of bacterial origin. In support of this claim we observed that TRAP1 protein (from Humans and *Drosophila*) is encoded on the nuclear genome but localizes to the mitochondria due to a mitochondrial localization sequence in their N-terminus [18]. In some instances, TRAP homologues have also been found to localize to the cytosol or the nucleus [39,40]. On the other hand, phylogenetic analyses of mitochondrial genes including both small and large subunit *rRNA *and *Cob *and *Cox1*, suggested that mitochondria monophyletically arose from within a-proteobacteria [41,42]. Our phylogeny suggests that TRAP is monophyletic, and its sister lineage is a sequence from *Desulfitobacterium hafniense *a Fimicutes bacterium, not a a-proteobacteria. In Bacteria Group A, Fimicutes proteins are sister proteins to both the TRAP clade of proteins and to the HSP90A+B+C clade, which suggests a gene duplication event happened early in Group A (Fig 5B). TRAP proteins are most similar to eukaryote HTPG, both lacking Variable regions B and D. The N-terminal transit peptide sequence for targeting TRAP to mitochondria, not present in HTPG proteins, appears to have evolved at the base of the TRAP lineage. TRAP genes only exist in Animalia and some Alveolata and Englenozoa species in Protista, and appear to be absent in others (Plantae, Fungi and some species in Protista). The simplest way of explaining this pattern is for TRAP proteins to have evolved in a basal Protista lineage, which eventually gave rise to other Protista, to Animalia, and to Fungi, and for these proteins to have subsequently been lost in the Fungi lineage.

### HSP90B subfamily of genes

Our data revealed that HSP90A+B+C is a monophyly with high bootstrap support and 3 signature motifs (Table 4), rooted also to Firmicutes HTPG genes but distant from eukaryote TRAP genes. We suggest that eukaryote HSP90A+B+C and TRAP originated from different Group A HTPG ancestor copies present in the common ancestor of Firmicutes and early eukaryotes. There are three main theories to explain the origin of eukaryotes. According to the archaeal hypothesis, a primitive amitochondriate eukaryote originated from an archaeabacterium [43]. The chimera hypothesis suggests that an amitochondriate cell emerged as a fusion between an archaeabacterium and a Gram-negative eubacterium, with their genomes having mixed in some way [44,45]. Cavalier-Smith [36,37] suggested that the Eukaryota and Archaea, as sisters, evolved from neomura, the suggested common ancestor that arose from within Actinobacteria. Our results reject the archaeal hypothesis due to the lack of a HSP90 homologue in Archaea, and also the chimera hypothesis as Firmicutes is a Gram-positive, not a Gram-negative bacteria. Because Firmicutes and Actinobacteria are both Gram-positive bacteria that share the closest HSP90 sequence to eukaryotic HSP90, our data support the neomura synthesis. However, we have to assume that archaebacteria originally had a HSP90 homologue that was subsequently lost. Our data show that all HSP90A+B+C and TRAP sequences share 2 signature motifs (Table 4) that originated within HTPG Group A. This suggests that HSP90A+B+C and TRAP stem from a possible gene duplication event early in the evolution of Group A, which is supported by a moderately high bootstrap. Both ancestral sequences were taken into the nuclear genome of an eukaryotic cell when eukaryotes originated. Firmicutes also has a Group C HTPG copy, but we could not determine whether a Group B HTPG copy also exists in this group of bacteria due to a limitation of sequence data. We assume that any HTPG copy other than those of Group A was not transferred into the nuclear genome of the original eukaryote cell.

Gupta [23] suggested that HSP90A (the cytosolic copy) and HSP90B (the copy that localizes to the ER) constitute paralogous gene families that arose by a gene duplication event that took place very early in the evolution of eukaryote cells. Only 22 HSP90A, 7 HSP90B and 1 HSP90C proteins sequences were involved in his phylogenetic study. Subsequently, Emelyanov [24] and Stechmann & Cavalier-Smith [17,27] included 9 and 57 HSP90A, 2 and 9 HSP90B and 2 and 3 HSP90C sequences, respectively, in their phylogenetic studies, and considered that HSP90B and HSP90C (the copy that localizes to the chloroplast) make a paraphyletic group, and sister group to HSP90A. These analyses, however, did not include any protist HSP90B sequences. Our sequence data (78 HSP90A, 31 HSP90B and 6 HSP90C) were selected from all sequences currently available in the databases, and represents the largest data set assembled for the gene family across the broadest range of organisms. Importantly, our data set contains 5 protist HSP90B sequences. In contrast to earlier conclusions, our analysis revealed that HSP90B originated earlier relative to HSP90C and HSP90A. The key-defining characteristic of all eukaryotic cells is the presence of a membrane-bounded nucleus. It is widely accepted that the eubacterial partner developed numerous membrane infolds in the formation of the eukaryote cell. The detachment of these membrane infolds from the symbiotic eubacteria eventually led to the creation of the ER and nuclear envelope [46]. The ER, a new compartment in the cell, now required molecular chaperones to transmit information within the compartment and to help transport "passenger proteins" across the membranes. Because both of these functions have been associated with HSP90B members [47], we propose that the ancestral HTPG of the partner eubacteria evolved into HSP90B at the very beginning of the eukaryote cell formation.

Subsequently, the genome of the eubacterial partner was transferred to the newly formed nucleus, which led to the creation of a new cell, the common ancestor of all eukaryotes. Subsequently, two gene duplication events took place that led to HSP90C and HSP90A, respectively. The nested origin of the HSP90A and HSP90C clades within the HSP90B sequences, makes this latter group a paraphyletic group. HSP90B is the most diverse eukaryote HSP90 subfamily, even lacking a unique signature motif. Its genes are glucose-regulated and induced by glucose starvation [1]. In addition, its proteins, once secreted to the ER, are also known to participate in protein folding and assembly, in protein secretion, in protecting cells from undergoing apoptosis, and in mediating immunogenicity in tumor and virus-infected cells [1,48]. Structurally, they have an ER transit peptide, their Variable region B is between HSP90A and HSP90C in length and their Variable region D is the longest of all HSP90 subfamilies. The highly conserved C-terminus sequence KDEL was thought to facilitate HSP90B retention in the ER; however, our data show that the motif is variable across organisms. Interestingly, HSP90B was thought to be absent in Fungi [17], but our genomic study detected a HSP90B copy in the fungus *Encephalitozoon cuniculi*, but not in *Cryptococcus neoformans, Neurospora crassa *and *Saccharomyces cerevisiae*, which implies that the HSP90B has been lost in several fungi lineages.

### HSP90C subfamily of genes

HSP90C genes appear to be monophyletic, given the high bootstrap support, and have 5 signature motifs. An earlier study [30] made use of only four available HSP90C sequences for *Arabidopsis thaliana*, *Chlamydomonas reinhardtii*, *Oryza sativa *and *Secale cereale*, respectively. All of them contain a DPW motif at their C-termini, and we named them HSP90C1. Surprisingly, our genomic study revealed an additional copy from *Arabidopsis thaliana *and *Oryza sativa*. The additional copies have high similarity to HSP90C1 but lack the DPW motif, and we named them HSP90C2. The *Chlamydomonas reinhardtii *genome only has the HSP90C1 copy [30], which is quite distinct from the HSP90C1 copy from higher plants. Our phylogeny shows that the single HSP90C copy present in *Chlamydomonas *duplicated into the HSP90C1 and HSP90C2 copies of higher plants, both clades supported with high bootstrap values. Due to the limitation of the sequences available, the signature sequences of HSP90C members and the exact origin of the subsequent gene duplication event need to be further addressed. HSP90C1 proteins are localized only in the chloroplast [49-51]. This organelle is universally accepted to have monophyletically arisen from within cyanobacteria [52-54]. The phylogeny of HSP60 and HSP70 also supported this synthesis [24]. In agreement with Emelyanov [24] and Stechmann & Cavalier-Smith [17], our data support the origin of chloroplast HSP90C from ER HSP90B and from Protista ancestors, not from plant HSP90B. This new HSP90C copy later acquired a chloroplast transit peptide. As in mitochondrial TRAP, the restricted presence of HSP90C genes, in this case only in plants and in some species of protista, implies that either HSP90C genes appeared in a lineage of protista that gave rise to plants or that they evolved earlier but have been lost in other lineages through unknown evolutionary pressures. Our low bootstrap values at the base of the HSP90C clade do not allow us to distinguish between these alternative explanations. HSP90C1 proteins of *Arabidopsis thaliana *and *Chlamydomonas reinhardtii *are constitutively expressed but they are also light and heat-shock inducible. They exhibit weak ATPase activity that can be inhibited by the HSP90-specific inhibitor radicicol, and possibly function in stress management and in the maturation of specific client protein, e.g. components of signal transduction pathways [50,51]. This is the first time that HSP90C2 genes have been reported, and their expression and function await to be addressed. Based on the high sequence similarity with HSP90C1, HSP90C2 proteins are possibly also working in the chloroplast but we cannot exclude the possibility that they are localized to other cell compartments

### HSP90A subfamily of genes

Our study indicates that HSP90A is also a monophyletic with high bootstrap support and 11 signature motifs. This is consistent with earlier studies in which all HSP90A sequences were clustered into a single clade [17,23,24,27]. In vertebrates HSP90A was divided into two classes HSP90AA and HSP90AB, with 7 and 1 signature sequences, respectively. However, the difference between these two classes was markedly smaller than the difference among HSP90A sequences across animals, fungi, plant and protists. An additional gene duplications event is required to explain the presence of HSP90AA and HSP90AB, which took place early in the origin of vertebrates. Structurally, HSP90A lacks the signal peptide in its N-terminus and its Variable region B is the longest of all subfamilies. HSP90A proteins are largely cytosolic [1], have ATPase activity, and are involved in the folding of cell regulatory proteins and the re-folding of stress-denatured polypeptides [33]. HSP90AA is somewhat inducible, whereas HSP90AB1 is more constitutively expressed [1].

### Organismal evolution

The unicellular protists are universally accepted to be the original sources of eukaryote diversity. Based primarily on their morphology and biology, protists can be assigned to several dozen well-characterized groups [55]. Each of the other eukaryote kingdoms (Animalia, Fungi, Plantae) can be directly traced back to a corresponding protist origin [37,38,56]. This explains why protist sequences are at the base of the various organismal branches within our HSP90A clade.

Many attempts have been made to establish a natural, phylogenetic system of eukaryotes, but the relationships and the order of evolutionary emergence of many diverse groups remain unresolved, primarily because of the lack of clear synapomorphies [57]. Our phylogeny of HSP90A among Animalia, Fungi, Plantae and Protista do not seem completely consistent with the current view of eukaryote evolution that postulates that Animalia has a closer relationship to Fungi than to Plantae [36,37,56]. This contradiction with respect to the branching order was not supported by strong bootstrap values and was probably brought about by the limited number of genomes analyzed within the eukaryotes. Phylogenies of other genes, including HSP70, GRP78 and 16S rRNA [44,58], and 22 protein-coding genes [57] place Diplomonadida (containing *Giardia lamblia*) and Parabasala at the base of the Eukaryota. Our phylogeny of the HSP90A clade also clearly demonstrates that *Giardia lamblia *is basal to all eukaryotes, followed by *Trichomonas vaginalis *in Parabasala.

## Conclusion

The present study presents the first comparative genomic study and evolutionary analysis of the HSP90 family of genes across all kingdoms of organisms. HSP90 family members underwent multiple duplications and also subsequent losses during their evolution. Based on the phylogeny of their proteins and the cell compartments the proteins function in, we divided the gene family into 5 subfamilies, HSP90A, HSP90B, HSP90C, TRAP, and HTPG, and established a new nomenclature system. This system has not been used for other gene families, and it may serve as a model for the nomenclature of other chaperone gene families (e.g. HSP100, HSP70, HSP60, and small HSP) that have a similar status to the HSP90 gene family. This study established an overall framework of information for the family of HSP90 genes, which may facilitate and stimulate the study of this gene family across all organisms.

## Methods

### Sequence retrieving and genome screening

In order to find all putative HSP90 family members we performed PSI-BLAST [59] searches of the protein database throughout all organisms at NCBI [60] using *Homo sapiens *protein sequences HSP90AA1 (NP_005339.2; [28]), HSP90AB1 (NP_031381.2), HSP90B1 (NP_003290.1), TRAP1 (NP_057376.1), *Arabidopsis thaliana *HSP90C1 (AAD32922.1), and *Escherichia coli *HTPG (AAA23460.1) as queries, respectively. Each search resulted in a list of similar sequences, which was added to the next round of PSI-BLAST iteration searches, and each search continued until no new sequence with an alignment score above the default threshold was retrieved. The sequences returned by these queries were combined and all redundant sequences were discarded. All sequences were examined individually and aligned using Clustal X [61]. Any sequence with sequence identity lower than a predefined threshold for assigning homology [62] was excluded from the study. Only complete and representative protein sequences were employed for subsequent evolutionary study, but remaining sequences were also used for the comparative analysis of sequence conservation. Sequence identity and similarity were calculated using BioEdit v5.0 [63].

Thirty-two species of nuclear genomes were investigated belonging to Archaea (1 species), Bacteria (10), Fungi (4), Protista (4), Plantae (3) and Animalia (10) (Table 1, Table 2). To localize the members of the HSP90 gene family within each genome, we used MapViewer at NCBI. A TBLASTN search against each genome assembly was applied using each of the protein query sequences mentioned above, whereas a BLASTN search was performed with the nucleotide query sequences. Subsequently, a 200 kb genome sequence flanking each hit, or close hits, was downloaded from the corresponding strand of the chromosome. The GENSCAN software [64] was used to identify the genes in each 200kb sequence. In order to reduce prediction error in genome research, we filtered out predictions of gene sequences with an exon probability less than 50% in the GENSCAN calculation, and/or with a coding region (CDS) shorter than 200 bp. DNA and protein sequences of the predicted genes were separately aligned with the sequences of the six queries mentioned above, to remove genes other than those in the HSP90 family using the same threshold for assigning homology [62]. All mRNA sequences retrieved from the databases, and coding sequences predicted from each species, were run on MapViewer to obtain their chromosomal locations according to corresponding Map Elements from the search results. All different mRNA sequences and predicted coding sequences that mapped to the same chromosomal site were considered sources from a single gene, and a single mRNA or predicted coding sequence most closely matching the genomic and query sequences was selected to represent the gene. The nucleotide starting and ending locations of introns and exons of the selected transcript were recorded in order to identify total exon numbers.

### Protein sequence properties

The signal peptide was predicted using both neural network (NN) and hidden markov model (HMM) methods with the program SignalP v3.0 [65,67]. The size of the mature proteins was estimated as the size of precursors after removal of the predicted signal peptide. The molecular mass of the precursor and mature protein was calculated using BioEdit. In order to identify biologically significant motifs and domains for each divergent protein sequence we used two different software programs. ScanProsite software [66] was used to search against the PROSITE database of protein families and domains (Release 18.40 of 22-Nov-2004) [68], whereas InterProScan [69] was used against InterPro, a database of protein domains and functional sites [70]. In the genome study, pseudogenes were determined by the absence of both the transcript and the functional domain in the predicted sequence. In order to characterize the HSP90 family of proteins and its subdivided classes, the amino acid residues completely conserved throughout the family and in each subfamily, and the conserved/variable regions, were identified by BioEdit based on the alignments produced by Clustal X.

### Phylogenetic analysis

The phylogenetic analysis was performed on a.a. sequences using maximum parsimony with PAUP* v4.0b8 [71]. We performed a heuristic search employing step-wise addition with 1000 random taxon addition sequence replicates and best trees held at each step. Branches were collapsed if maximum branch length was zero. All characters were given equal weight and gaps were treated as "missing". The node support was assessed using 5000 bootstrap pseudo-replicates with step-wise addition and parsimony criteria.

## Abbreviations

a.a., amino acid; ER, endoplasmic reticulum; HTPG, high temperature protein G; HSP90, 90 kDa heat-shock protein; TRAP, tumor necrosis factor receptor-associated protein.

## Authors' contributions

BC designed and conducted the study on comparative genome analysis and evolution, performed database searches, sequence alignment, phylogenetic analysis, gene structure prediction and nomenclature, and drafted the manuscript. DZ participated in data analysis and manuscript revision. AM participated in research design and in the drafting of the manuscript. All authors read and approved the final manuscript

## Supplementary Material

Additional File 1 Supplementary Sequences. Nucleotide sequences of newly predicted genes, and deduced a.a. sequences from these sequences as well as from (OSA)HSP90C2 mRNA sequences available in database at NCBI. Click here for file

## Figures and Tables

**Table 1:** Summary of the HSP90 family of genes present on the genomes of 32 species, showing the number of functional and total (total in parenthesis) genes, exon number on genomes corresponding to in coding regions and mRNAs, and length and molecular mass of mature and precursor (in parenthesis) polypeptide in each subfamily member. Only the functional and full-length sequences are included in the calculation of exon number, length and molecular mass of polypeptide. The details are in Table 2.

**Group**	**Species**	**Total**	**HTPG**	**TRAP**	**HSP90C**	**HSP90B**	**HSP90A**	**HSP90AA**	**HSP90AB**
**ARCHAEA**	***Methanosarcina mazei***	1 (1)							
**BACTERIA**	***Bacillus subtilis***	1 (1)	1 (1)						
	***Bacteroides fragilis***	2 (2)	2 (2)						
	***Bordetella pertussis***	1 (1)	1 (1)						
	***Borrelia burgdorferi***	1 (1)	1 (1)						
	***Escherichia coli***	1 (1)	1 (1)						
	***Geobacter sulfurreducens***	1 (1)	1 (1)						
	***Gloeobacter violaceus***	1 (1)	1 (1)						
	***Helicobacter pylori***	1 (1)	1 (1)						
	***Streptomyces coelicolor***	2 (2)	2 (2)						
	***Wolbachia endosymbiont***	1 (1)	1 (1)						
**PROTISTA**	***Dictyostelium discoideum***	2 (2)				1 (1)	1 (1)		
	***Entamoeba histolytica***	4 (4)				1 (1)	3 (3)		
	***Plasmodium falciparum***	2 (2)				1 (1)	1 (1)		
	***Trypanosoma cruzi***	4 (4)				1 (1)	3 (3)		
**PLANTAE**	***Arabidopsis thaliana***	7 (7)			2 (2)	1 (1)	4 (4)		
	***Chlamydomonas reinhardtii***	3 (3)			1 (1)	1 (1)	1 (1)		
	***Oryza sativa***	7 (8)			2 (2)	1 (1)	4 (5)		
**FUNGI**	***Cryptococcus neoformans***	2 (2)					1 (1)		
	***Encephalitozoon cuniculi***	1 (1)				1 (1)	1 (1)		
	***Neurospora crassa***	1 (1)					1 (1)		
	***Saccharomyces cerevisiae***	2 (2)					2 (2)		
**ANIMALIA**	***Anopheles gambiae***	4 (5)		1 (1)		1 (1)	2 (3)		
	***Apis mellifera***	4 (4)		1 (1)		1 (1)	2 (2)		
	***Caenorhabditis elegans***	3 (3)		1 (1)		1 (1)	1 (1)		
	***Ciona intestinalis***	3 (4)		1 (1)		1 (2)	1 (1)		
	***Danio rerio***	6 (8)		1 (1)		2 (2)		2 (4)	1 (1)
	***Drosophila melanogaster***	3 (3)		1 (1)		1 (1)	1 (1)		
	***Drosophila pseudoobscura***	3 (3)		1 (1)		1 (1)	1 (1)		
	***Gallus gallus***	4 (4)		1 (1)		1 (1)		1 (1)	1 (1)
	***Homo sapiens***	5 (16)		1 (1)		1 (3)		2 (6)	1 (6)
	***Strongylocentrotus purpuratus***	4 (4)		1 (1)		1 (1)	2 (2)		
**Exon number on genome corresponding to coding regions and in mRNAs (mRNAs in parenthesis)**			1 (1) – 1(1)	3 (3) – 19 (19)	10 (?) – 21 (21)	1 (?) – 18 (18)	1 (1) – 11 (12)	8 (?) – 12 (12)	7 (7) – 11 (12)
**Length of mature and precursor polypeptide (precursor in parenthesis)**			588 (588) – 681 (681)	644 (688) – 687 (719)	756 (810) – 785 (811)	695 (711) – 800 (823)	689 (689) – 745 (745)	728 (728) – 854 (854)	724 (724) – 737 (737)
**Molecular mass of mature and precursor polypeptide precursor (in parenthesis) (kDa)**			66.7 (66.7) – 78.0 (78.0)	74.8 (78.1) – 77.9 (81.5)	84.2 (89.3) – 89.0 (91.5)	79.5 (81.2) – 91.5 (94.2)	78.3 (78.3) – 86.2 (86.2)	84.1 (84.1) – 98.1 (98.1)	83.3 (83.3) – 84.8 (84.8)

**Table 2:** HSP90 family of genes present on the genomes of 32 species, and their chromosomal location, intron/exon structure, molecular mass, signal peptide and prediction of genes that are functional.

Gene name^a^	Accession number^b^		Chrom. location^c^	Exon no^d^	a.a.length^e^	Mass (kDa)^f^	Function^g^
						
	mRNA	a.a.					
**ARCHAEA**							
***Methanosarcina mazei***							
(MMA)NP_634445.1	NC_003901.1	NP_634445.1	N/A+	1	982	114.6	F
**BACTERIA**							
***Streptomyces coelicolor***							
(SCO)HTPG1	NC_003888.3	NP_631561.1	N/A+	1	638	71.6	F
(SCO)HTPG2	NC_003888.3	NP_625688.1	N/A-	1	615	66.7	F
***Bacteroides fragilis***							
(BFR)HTPG1	NC_006347.1	YP_099692.1	N/A-	1	681	78.0	F
(BFR)HTPG2	NC_006347.1	YP_097520.1	N/A+	1	588	67.3	F
***Gloeobacter violaceus***							
(GVI)HTPG1	NC_005125.1	NP_924760.1	N/A+	1	614	70.3	F
***Bacillus subtilis***							
(BSU)HTPG1	NC_000964.2	NP_391861.1	N/A-	1	626	72.3	F
***Wolbachia endosymbiont***							
(WEN)HTPG1	NC_002978.6	NP_966987.1	N/A+	1	635	72.7	F
***Bordetella pertussis***							
(BPE)HTPG1	NC_002929.2	NP_878979.1	N/A+	1	635	71.2	F
***Geobacter sulfurreducens***							
(GSU)HTPG1	NC_002939.4	NP_953436.1	N/A+	1	650	73.7	F
***Helicobacter pylori***							
(HPY)HTPG1	NC_000921.1	NP_222917.1	N/A+	1	621	71.1	F
***Escherichia coli***							
(ECO)HTPG1	NC_000913.2	NP_415006.1	N/A+	1	624	71.4	F
***Borrelia burgdorferi***							
(BBU)HTPG1	NC_001318.1	NP_212694.1	N/A-	1	650	75.4	F
**FUNGI**							
***Saccharomyces cerevisiae***							
(SCE)HSP90A1	K01387.1	AAA02743.1	16-	1 (1)	709	81.4	F
(SCE)HSP90A2	M26044.1	AAA02813.1	13+	1 (1)	705	80.9	F
***Cryptococcus neoformans***							
(CNE)HSP90A1	XM_568451.1	XP_568451.1	13-	6	700	79.3	F
(CNE)HSP90B1	XM_571124.1	XP_571124.1	5+	11	758 (780, H)	85.5 (87.8)	F
***Encephalitozoon cuniculi***							
(ECU)HSP90A1	NC_003229.1	NP_584635.1	2+	1	690	79.0	F
***Neurospora crassa***							
(NCR)HSP90A1	AABX01000271.1	EAA32062.1	5+	2	705	80.2	F
**PROTISTA**							
***Plasmodium falciparum***							
(PFA)HSP90A1	NC_004328.1	NP_704028.1	7+	2	745	86.2	F
(PFA)HSP90B1	NC_004316.1	NP_701576.1	12-	1	793 (821, H)	91.6 (95.0)	F
***Dictyostelium discoideum***							
(DDI)HSP90A1	AAFI01000009.1	EAL73152.1	1-	3	700	79.9	F
(DDI)HSP90B1	AAFI01000066.1	EAL67255.1	3-	2	739 (767, H)	84.1 (87.1)	F
***Entamoeba histolytica***							
(EHI)HSP90A1	AAFB01000333.1	EAL47778.1	(Sca 00092+)	1	718	83.0	F
(EHI)HSP90A2	AAFB01000334.1	EAL47746.1	(Sca 00092+)	1	718	83.0	F
(EHI)HSP90A3	AAFB01000781.1	EAL44230.1	(Sca 00284+)	2	702	81.1	F
(EHI)HSP90B1	AAFB01000726.1	EAL44584.1	(Sca 00256-)	1	695 (711, H)	79.5 (81.2)	F
***Trypanosoma cruzi***							
(TCR)HSP90A1	AAHK01000382.1	EAN93041.1	N/A	1	704	80.7	F
(TCR)HSP90A2	AAHK01000690.1	EAN89940.1	N/A	1	704	80.7	F
(TCR)HSP90A3	AAHK01002401.1	EAN82629.1	N/A	1	704	80.7	F
(TCR)HSP90B1	AAHK01000131.1	EAN96800.1	N/A	1	740 (762, H)	84.7 (87.0)	F
**PLANTAE**							
***Arabidopsis thaliana***							
(ATH)HSP90A1	NM_124642.2	NP_200076.1	5+	4 (4)	705	81.2	F
(ATH)HSP90A2	NM_124985.3	NP_200414.1	5-	3 (3)	699	80.1	F
(ATH)HSP90A3	NM_124983.3	NP_200412.1	5+	3 (3)	699	80.0	F
(ATH)HSP90A4	NM_124982.2	NP_200411.1	5+	3 (3)	699	80.1	F
(ATH)HSP90B1	NM_118552.2	NP_194150.1	4-	15 (15)	800 (823, H)	91.5 (94.2)	F
(ATH)HSP90C1	NM_126439.2	NP_178487.1	2+	19 (19)	756 (780, H)	86.2 (88.7)	F
(ATH)HSP90C2	NM_111656.2	NP_187434.1	3+	21 (21)	778 (803, H)	88.2 (91.0)	F
***Oryza sativa***							
(OSA)HSP90A1^P^	XM_483191.1	XP_483191.1	8-	3	699	80.2	F
(OSA)HSP90A2^P^	XM_470993.1	XP_470993.1	4+	3	703	80.2	F
(OSA)HSP90A3^P^	AP005392.3	BAD33406.1	9-	1	699	80.2	F
(OSA)HSP90A4^P^	AP005392.3	BAD33409.1	9-	1	699	80.2	F
(OSA)HSP90A5P^NP^	(OSA)HSP90A5N^N^*	(OSA)HSP90A5P^N^*	9-	1	437*		P
(OSA)HSP90B1	AB037681.1	BAA90487.1	6-	15 (15)	791 (810, H)	90.6 (92.8)	F
(OSA)HSP90C1	XM_483065.1	XP_483065.1	8-	19 (19)	763 (785, H)	86.3 (88.3)	F
(OSA)HSP90C2	AK073817.1	(OSA)HSP90C2P^N^	9-	14 (17)	785 (811, H)	89.0 (91.5)	F
***Chlamydomonas reinhardtii***							
(CRE)HSP90A1	Schroda (2004)		(Sca 2397)	8	705	80.7	F
(CRE)HSP90B1	Schroda (2004)*		(Sca 2084, 1758)	≥11	736*		F
(CRE)HSP90C1	AY705371.1	AAU10511.1	(Sca 6)	10	756 (810, H)	84.2 (89.3)	F
**INVERTEBARATE**							
***Drosophila melanogaster***							
(DME)HSP90A1	NM_079175.2	NP_523899.1	3L+ 63B11	1 (2)	717	81.9	F
(DME)HSP90B1	NM_143344.2	NP_651601.1	3R+ 98B6	5 (5)	767 (787, H)	88.1 (90.2)	F
(DME)TRAP1	NM_058091.3	NP_477439.2	2R- 42C1	3 (3)	673 (691, N)	74.7 (78.0)	F
***Drosophila pseudoobscura***							
(DPS)HSP90A1^P^	NM_079175.2	NP_523899.1	XR-	1	717	81.8	F
(DPS)HSP90B1^P^	CM000070.1	EAL27390.1	2-	5	772 (792, H)	88.4 (90.5)	F
(DPS)TRAP1^P^	CM000071.1	EAL26542.1	3-	3	644 (688, N)	74.8 (78.1)	F
***Anopheles gambiae***							
(AGA)HSP90A1^P^	XM_308800.2	XP_308800.2	2L-	2	689	78.3	F
(AGA)HSP90A2^P^	XM_308799.1	XP_308799.1	2L+	3	720	82.1	F
(AGA)HSP90A3P^NP^	(AGA)HSP90A3N ^N^*	(AGA)HSP90A3P^N^*	2L+	2	380*		P
(AGA)HSP90B1^NP^	(AGA)HSP90B1N ^N^	(AGA)HSP90B1P^N^	2R-	2	778 (800, H)	88.9 (91.3)	F
(AGA)TRAP1^NP^	(AGA)TRAP1N ^N^	(AGA)TRAP1P^N^	3L+	3	677 (713, H)	76.8 (80.6)	F
***Apis mellifera***							
(AME)HSP90A1^P^	XM_392456.2	XP_392456.2	(Group 7-)	3 (3)	700	80.7	F
(AME)HSP90A2^P^	XM_395168.2	XP_395168.2	(Group 1-)	3 (3)	699	80.5	F
(AME)HSP90B1^P^	XM_395614.2	XP_395614.2	(Group 8-)	5 (5)	766 (786, H)	88.5 (90.8)	F
(AME)TRAP1^P^	XM_623363.1	XP_623366.1	(Group 6-)	13 (13)	668 (693, N)	76.7 (79.6)	F
***Caenorhabditis elegans***							
(CEL)HSP90A1	NM_074225.2	NP_506626.1	5+	4 (5)	702	80.2	F
(CEL)HSP90B1	NM_069679.2	NP_502080.1	4+	5 (5)	737 (760, H)	84.6 (87.1)	F
(CEL)TRAP1	NM_171188.2*	NP_741219.1*	3-	9 (9)	464*		F
***Strongylocentrotus purpuratus***							
(SPU)HSP90A1	XM_777937.1*	XP_783030.1*	N/A-	≥ 8 (9)	510*		F
(SPU)HSP90A2	XM_782798.1*	XP_787891.1*	N/A+	≥ 5	319*		F
(SPU)HSP90B1	NM_214643.1	NP_999808.1	N/A+	17	784 (806, H)	90.0 (92.3)	F
(SPU)TRAP1	XM_778412.1	XP_783505.1	N/A+	15	665 (691, N)	75.5 (78.6)	F
**VERTEBRATE**							
***Homo sapiens***							
Chen et al.A (2005)							
***Gallus gallus***							
(GGA)HSP90AA1	X07265.1	CAA30251.1	5-	8	728	84.1	F
(GGA)HSP90AB1	XM_444655.1	XP_444655.1	3-	11	725	83.5	F
(GGA)HSP90B1	NW_060209.1	NP_989620.1	1-	18	774 (795, H)	89.5 (91.6)	F
(GGA)TRAP1	NW_060530.1	XP_414963.1	14-	18	663 (699, N)	75.5 (79.5)	F
***Danio rerio***							
(DRE)HSP90AA1	AF068773.1	AAC21567.1	20-	10 (10)	726	83.6	F
(DRE)HSP90AA2^NP^	(DRE)HSP90AA2N^N^	(DRE)HSP90AA2P^N^	20-	10	734	84.7	F
(DRE)HSP90AA3P^NP^	(DRE)HSP90AA3N^N^*	(DRE)HSP90AA3P^N^*	7+	12	838*		P
(DRE)HSP90AA4P^NP^	(DRE)HSP90AA4N^N^*	(DRE)HSP90AA4P^N^*	7+	3	417*		P
(DRE)HSP90AB1	AF042108.1	AAB96969.1	7-	7 (7)	725	83.4	F
(DRE)HSP90B1	BC063951.1	AAH63951.1	4+	18 (18)	772 (793, H)	88.9 (91.3)	F
(DRE)HSP90B2^NP^	(DRE)HSP90B2N^N^	(DRE)HSP90B2P^N^	4+	18	773 (794, H)	89.1 (91.4)	F
(DRE)TRAP1^NP^	(DRE)TRAP1^N^*	(DRE)TRAP1^N^*	23+	≥8	414*		F
***Ciona intestinalis***							
(CIN)HSP90A1	AK115284.1	AK115284.1P^N^	(Sca 118+)	11 (12)	726	83.3	F
(CIN)HSP90B1^NP^	(CIN)HSP90B1N^N^*	(CIN)HSP90B1P^N^*	(Sca 963-)	≥15	732*		F
(CIN)HSP90B2P^NP^	(CIN)HSP90B2N^N^*	(CIN)HSP90B2P^N^*	(Sca 462-)	≥2	119*		P
(CIN)TRAP1^NP^	(CIN)TRAP1^N^	(CIN)TRAP1^N^	(Sca 143+)	13	687 (719, N)	77.9 (81.5)	F

^a P ^predicted by genome annotation; ^NP ^predicted in this study. The abbreviations of species names in parenthesis are in Table 3. The genes with their nomenclatures ended with "P" are putative pseudogenes.

^bN ^new sequences identified/deduced in this study and available at Additional File 1. The sequences marked with "*" are not full-length.

^c ^Chromosomal location. + forward strand; – reverse strand. The numbers/letters are the chromosome numbers/letters, and N/A indicates unavailability of the chromosome number and Sca indicates Scaffold.

^d ^Total number of exons in coding regions. The numbers in parenthesis include exons in untranslated regions.

^e ^length of mature polypeptide with that of precursor and prediction method (Hidden Markov models, H; Neural networks, N) in parenthesis. The sequences marked "*" are incomplete, or from putative pseudogenes, and their mature polypeptides are not predicted.

^f ^molecular mass of mature polypeptide with that of precursor in parenthesis.

^g ^F – functional genes predicted by transcript and domain identification. P – putative pseudogenes.

**Table 3:** The name abbreviation of 146 species containing HSP90 family members that were analyzed in the phylogenetic study.

**ARCHAEA (1):** *Methanosarcina mazei*: MMA **BACTERIA (59):** *Bacillus subtilis*: BSU *Bacteroides fragilis*: BFR *Bdellovibrio bacteriovorus*: BBA *Bordetella pertussis*: BPE *Borrelia burgdorferi*: BBU *Bradyrhizobium japonicum*: BJA *Buchnera aphidicola*: BAP *Burkholderia fungorum*: BFU *Campylobacter jejuni*: CJE *Chlorobium tepidum*: CTE *Chloroflexus aurantiacus*: CAU *Chromobacterium violaceum*: CVI *Clostridium perfringens*: CPE *Corynebacterium glutamicum*: CGL *Coxiella burnetii*: CBU *Cytophaga hutchinsonii*: CHU *Desulfitobacterium hafniense*: DHA *Desulfovibrio desulfuricans*: DDE *Desulfovibrio vulgaris*: DVU *Escherichia coli*: ECO *Fusobacterium nucleatum*: ENU *Geobacter sulfurreducens*: GSU *Gloeobacter violaceus*: GVI *Haemophilus influenzae*: HIN *Helicobacter hepaticus*: HHE *Helicobacter pylori*: HPY *Leptospira interrogans*: LIN *Listeria monocytogenes*: LMO *Mesorhizobium loti*: MLO *Mycobacterium leprae*: MLE *Mycobacterium tuberculosis*: MTU *Nitrosomonas europaea*: NEU *Nostoc punctiforme*: NPU *Oceanobacillus iheyensis*: OIH *Parachlamydia *sp.: PSP *Pasteurella multocida*: PMU *Porphyromonas gingivalis*: PGI *Prochlorococcus marinus*: PMA *Pseudomonas aeruginosa*: PAE *Pseudomonas syringae*: PSY *Ralstonia solanacearumgi*: RSO *Rhodopseudomonas palustris*: RPA *Rickettsia conorii*: RCO *Salmonella enterica*: SEN *Shewanella oneidensis*: SON *Sinorhizobium meliloti*: SME *Streptomyces coelicolor*: SCO *Thermobifida fusca*: TFU	*Thermosynechococcus elongates*: TEL *Treponema denticola*: TDE *Treponema pallidumgi*: TPA *Trichodesmium erythraeum*: TER *Vibrio vulnificus*: VVU *Wigglesworthia glossinidia*: WGL *Wolbachia endosymbiont*: WEN *Wolinella succinogenes*: WSU *Xanthomonas axonopodis*: XAX *Xylella fastidiosa*: XFA *Yersinia pestis*: YPE **PROTISTA (23)** *Achlya ambisexualis*: AAM *Babesia bovis*: BBO *Cyanidioschyzon merolae*: CME *Crypthecodinium cohnii*: CCO *Cryptosporidium hominis*: CHO *Cryptosporidium parvum*: CPA *Dictyostelium discoideum*: DDI *Eimeria tenella*: ETE *Entamoeba histolytica*: EHI *Giardia intestinalis*: GIN *Guillardia theta*: GTH *Leishmania infantum*: LIN *Leishmania major*: LMA *Plasmodium chabaudi*: PCH *Plasmodium falciparum*: PFA *Plasmodium yoelii yoelii*: PYO *Tetrahymena pyriformis*: TPY *Theileria annulata*: TAN *Theileria parva*: TPA *Toxoplasma gondii*: TGO *Trichomonas vaginalis*: TVA *Trypanosoma brucei*: TBR *Trypanosoma cruzi*: TCR **PLANTAE (13):** *Arabidopsis thaliana*: ATH *Catharanthus roseus*: CRO *Chlamydomonas reinhardtii*: CRE *Hevea brasiliensis*: HBR *Hordeum vulgare*: HVU *Ipomoea nil*: INI *Lycopersicon esculentum*: LES *Nicotiana benthamiana*: NBE *Oryza sativa*: OSA *Secale cereale*: SCE *Triticum aestivum*: TAE *Xerophyta viscose*: XVI *Zea mays*: ZMA	**FUNGI (13):** *Ashbya gossypii*: AGO *Aspergillus nidulans*: ANI *Candida albicans*: CAL *Candida glabrata*: CGL *Cryptococcus neoformans*: CNE *Encephalitozoon cuniculi*: ECU *Kluyveromyces lactis*: KLA *Neurospora crassa*: NCR *Paracoccidioides brasiliensis*: PBR *Podospora anserine*: PAN *Saccharomyces cerevisiae*: SCE *Schizosaccharomyces pombe*: SPO *Yarrowia lipolytica*: YLI **ANIMALIA (37):** *Anopheles albimanus*: AAL *Anopheles gambiae*: AGA *Antheraea yamamai*: AYA *Apis mellifera*: AME *Astyanax mexicanus*: ASM *Bemisia tabaci*: BTA *Bombyx mori*: BMO *Bos taurus*: BTA *Brugia pahangi*: BPA *Caenorhabditis briggsae*: CBR *Caenorhabditis elegans*: CEL *Chiromantes haematocheir*: CHA *Chlamys farreri*: CFA *Ciona intestinalis*: CIN *Danio rerio*: DRE *Delia antiqua*: DAN *Dendronephthya klunzingeri*: DKL *Dicentrarchus labrax*: DLA *Drosophila melanogaster*: DME *Drosophila pseudoobscura*: DPS *Eptatretus stoutii*: EST *Gallus gallus*: GGA *Heterodera glycines*: HGL *Homo sapiens*: HSA *Mus musculus*: MMU *Oncorhynchus mykiss*: OMY *Oncorhynchus tshawytscha*: OTS *Opistophthalmus carinatus*: OPI *Paralichthys olivaceus*: POL *Pongo pygmaeus*: PPY *Rattus norvegicus*: RNO *Salmo salar*: SSA *Schistosoma japonicum*: SJA *Schistosoma mansoni*: SMA *Spodoptera frugiperda*: SFR *Strongylocentrotus purpuratus*: SPU *Xenopus laevis*: XLA

**Table 4:** Signature sequences detected in each subfamily or in particular subfamily combinations. The numbers in parenthesis following the signature sequence indicate the residue numbers in the corresponding reference sequence. HSP90B has no unique signature sequence but the combination of these conserved motifs can distinguish it from other family members. The alignment is in Figure 1.

Subfamily	Signature sequence	Reference
HSP90AA	LIP (80–82), GDKKKK (272–277), TSASG (467–471), VSLKDYCT (475–482), QEEKK (561–565), EDD (701–703), MPP (717–719)	(HSA)HSP90AA1
HSP90AB	LKID (residues 71–74)	(HSA)HSP90AB1
HSP90A	SLIINT (31–36), NNLGTIA (105–111), SMIGQFGVGFYS (129–140), AGG (166–168), RGT (182–184), KHFSVEGQLEF (327–337), VKK (417–419), HED (450–452), TGE (495–497), IDEY (525–528), QALRD (617–621)	(HSA)HSP90AA1
HSP90B	FLREL (residues 100–104), IGQFGVGFYS (191–201), LPLNVSRE (442–449)	(HSA)HSP90B1
HSP90C	YQAEV (81–85), EVFLREL (101–107), LIGQFGVGFYSAFLV (193–207), DPLA (361–364), LYVKRVFISD (403–412), PRYL (420–423), DLPLNVSREILQESRIVR (434–451), APF (548–560)	(ATH)HSP90C1
TRAP	LLDIVA (97–102), NLGTIARSGS (171–180), IIGQFGVGFY (197–206), PLNLSRE (397–403), SPY (511–513)	(HSA)TRAP1
HTPG	No signature sequence	
HSP90A+B	VRRV (365–368), GMT (97–99)	(HSA)HSP90AA1
HSP90B+C	LPLNVSRE (442–449)	(HSA)HSP90B1
HSP90A+B+C	FLREL (44–48), VDS (389–391), LPLN (394–397)	(HSA)HSP90AA1
HSP90A+B+C+TRAP+Group A	IGQFGVGFY (131–139), PLN (395–397)	(HSA)HSP90AA1
HSP90A+B+C+TRAP+HTPG	Y (38), REL (46–48), N (51), D (54), G (95), G (97), GQFG (132–135), G (183), P (379), SRE (399–401)	(HSA)HSP90AA1
