# Supplementary material for: Comparative genomics and evolution of the HSP90 family of genes across all kingdoms of organisms
Source: BMC Genomics. 2006 Jun 17;7:156. doi: 10.1186/1471-2164-7-156 (PMC1525184; doi:10.1186/1471-2164-7-156)
Supplement: Additional File 1 — Supplementary Sequences. Nucleotide sequences of newly predicted genes, and deduced a.a. sequences from these sequences as well as from (OSA)HSP90C2 mRNA sequences available in database at NCBI. [file 1471-2164-7-156-S1.pdf]

Supplementary Sequences – Nucleotide sequences of newly predicted genes, and deduced a.a. sequences from these sequences as well as from (OSA)HSP90C2 mRNA sequences available in database at NCBI.

>(OSA)HSP90A5N
ATGAACAAGCAGAAACCCATTTGGTTGAGGAAGCCAGAGGAGATCACCAAGGAAGAGTACGCTGCTTTCTACAAGAG
CTTGACAAACGACTGGGAGGAACATCTGGCTGTCAAGCACTTCTCTGTGGAGGGTCAGCTTGAATTCAAGGCCATCC
TGTTTGTACCAAAGAGAGCGCCATTTGACCTCTTTGACACCAGGAAGAAGCAAAACAACATCAAGCTGTACGTACGC
CGGGTGTTTATCATGGACAACTGTGAGGAGTTGATCCCAGAGTGGCTCAGCTTTGTCAAGGGCATTGTTGATTCTGA
AGACCTTCCCCTCAACATCTCACGTGAGATGCTCCAGCAGAACAAGATCCTGAAGGTGATCCGCAAGAACCTTGTGA
AGAAGTGCGTGGAGCTCTTCTTTGAGATCGCTGAGAACAAGGAAGACTACAACAAGTTCTACGAGGCTTTCTCCAAG
AATCTCAAGCTTGGCATCCATGAGGATTCCACCAACAGGACCAAGATTGCTGAGCTCCTGAGGTATCACTCCACCAA
GAGTGGTGATGAGTTGACCAGCCTCAAGGACTACGTAACCCGGATGAAGGAGGGCCAGAGTGAGATCTATTACATCA
CTGGTGAGAGCAAGAAGGCTGTTGAGAACTCCCCCTTCCTCGAGAAGCTGAAGAAGAAGGGTTATGAGGTCCTGTAC
ATGGTTGATGCCATTGATGAGTACGCTGTTGGTCAGCTTAAGGAGTTTGAAGGCAAGAAGCTCGTCTCTGCCACCAA
GGAAGGTCTGAAGCTTGATGAGAGTGAGGACGAGAAGAAGCGGCAGGAGGAACTCAAGGAGAAGTTCGAGGGTCTGT
GCAAGGTGATCAAGGAGGTGCTCGGTGACAAGGTGGAGAAGGTTGTTGTCTCTGACCGTGTGGTGGACTCTCCCTGC
TGTCTAGTCACTGGGGAGTATGGCTGGACTGCTAACATGGAGAGGATCATGAAGGCCCAGGCTCTGAGGGACTCCAG
CATGGCCGGCTACATGTCTAGCAAGAAGACCATGGAGATCAACCCGGAGAATGCCATCATGGACGAGCTCCGCAAGC
GTGCCGATGCGGACAAGAATGACAAGTCCGTGAAGGACCTGGTGATGCTGCTCTTCGAGACTGCCCTGCTGACCTCC
GGTTTCAGCTTGGAGGACCCCAACACCTTCGGCACCAGGATCCACCGCATGCTCAAGCTCGGCCTGAGCATCGACGA
GGACGAGTCCGCCGAGGCTGACGCCGACATGCCGCCGCTGGAGGACGACGCCGGCGAGAGCAAGATGGAGGAGGTCG
ACTAA
>(OSA)HSP90A5P
MNKQKPIWLRKPEEITKEEYAAFYKSLTNDWEEHLAVKHFSVEGQLEFKAILFVPKRAPFDLFDTRKKQNNIKLYVR
RVFIMDNCEELIPEWLSFVKGIVDSEDLPLNISREMLQQNKILKVIRKNLVKKCVELFFEIAENKEDYNKFYEAFSK
NLKLGIHEDSTNRTKIAELLRYHSTKSGDELTSLKDYVTRMKEGQSEIYYITGESKKAVENSPFLEKLKKKGYEVLY
MVDAIDEYAVGQLKEFEGKKLVSATKEGLKLDESEDEKKRQEELKEKFEGLCKVIKEVLGDKVEKVVVSDRVVDSPC
CLVTGEYGWTANMERIMKAQALRDSSMAGYMSSKKTMEINPENAIMDELRKRADADKNDKSVKDLVMLLFETALLTS
GFSLEDPNTFGTRIHRMLKLGLSIDEDESAEADADMPPLEDDAGESKMEEVD
>(OSA)HSP90C2P
MLGASRRSVCTAAAAAAAGSRRRAAAGVASAVSGDSSVSSSSAPPRSVINGEPGVPQLQKRLLSVLAAPKLTGTDNA
ASLKLREGSLVGRRYESSAAAVDSSDTPPVEKHEYQAEVNRLMDLIVHSLYSNKEVFLRELVSNASDALDKLRYLSV
TDPDLIKDGAGLDIRIQTDKENGIITITDTGIGMTRQELVDSLGTIASSGTAKFLKALKESQEAGVDSNLIGQFGVG
FYSAFLVSDKVAVSTKSPKSDKQYVWEGEAESSSYTIREETDPEKLLPRGTRLTLYLKREDKGFAHPEKIQKLVKNY
SQFVSFPIYTWQEKGYTKEVEVDEPVEAKEGDDETKTEVKKKTKTVVEKYWDWELTNETQPIWLRNPREVSTEEYNE
FYKKTFNEYMDPLASSHFTTEGEVEFRSILYVPATKKEDITDRKTKNIRLYVKRVFISDDFDGELFPRYLSFVRGVV
DSNDLPLNVSREILQESRIVRIMRKRLVRKAFDMILGISCSENRDDYERFWENYGKFLKLGCMEDKENHKRIAPLLR
FLSSQSNEELISLDEYVENMKPEQKDIYYIAADSLSSAKHAPFLEKLNEKEYEVLFLVDPMDEVSVTNLNSYKDKRF
VDISKEDLDLGDKNEEKEKEIKQEYSQTCDWIKKRLGEKVARVDISNRLSSSPCVLVAAKFGWSANMERLMRAQSMG
DMSSLDFMRSRKVFEINPEHEIIKGLNAACRANPDDPEALKAVDILFETSMISSGFTPDNPAELSGKMYEMMSTAMA
SKWASHAQPAEMNLQRDSPVSSEPIEAEVIEPELVDDSQKK
>(AGA)HSP90A3N
ATGCCGGAACCACAAGAGGGCGAGACCTTCGCATTCCAGGCTGAGATTGCTCAGCTGATGTCCCTGATCATCAACAC
GTTCTACTCGAACAAGGAAATCTTCCTGCGTGAGTTGATCTCGAACTCGTCCGATGCGCTGGACAAGATCCGCTATG
AATCGCTGACGGATCCCTCGAAGCTGGAGTCGGGCAAGGAGCTGTTCATCAAGATCATCCCGAACAAGGAGGCCGGC
ACACTGACGCTGATCGATACCGGTATCGGCATGACGAAGGCCGACCTGGTGAACAACCTCGGTACGATTGCCAAGTC
GGGCACGAAGGCGTTCATGGAGGCGCTGCAGGCCGGCGCAGACATCAGCATGATCGGCCAGTTCGGTGTCGGTTTCT
ACTCGGCGTACCTGGTCGCCGACAAGGTGGTGGTGACGTCGAAGAACAACGACGACGAGCAGTACGTGTGGGAGTCG
TCGGCCGGCGGCTCGTTCACGGTGCGCCCGGACAGCGGGGAGCCGCTGGGCCGCGGTACCAAGATCGTGCTGCACAT
CAAAGGAGGACCAGCTGGAGTACCTGGAGGAGAGCAAGATCAAGCAGATCGTGAACAAGCACTCGCAGAGGACAAGG
CCAAGTTCGAGAACCTGTGCAAGGTGATGAAGTCGGTGCTGGAGAGCAAGGTGGAGAAGGTGATGGTGTCGAACCGG
CTGGTGGACTCGCCCTGCTGCATCGTGACGTCGCAGTACGGCTGGTCGGCCAACATGGAGCGCATCATGAAGGCGCA
GGCGCTGCGTGACTCGTCGGCCATGGGCTACATGGCGGGCAAGAAGCATCTCGAGATCAACCCGGACCATGCCATCA
TCGAGACGCTGCGCCAGCGCGCCGAGGCGGACAAGAACGATAAGGCGGTGAAGGATCTGGTGATTCTGCTGTTCGAG

ACGGCGCTGCTGTCCTCCGGCTTCTCGCTGGACGAGCCCGGAACCCATGCGTCCCGCATCTACCGCATGATCAAGCT
GGGTCTGGGCATCGACGAGGACGAGCCGATGACGACGGAGGACAGCAGCAGCGGTGCTGCGGCGGCGGCCCCAGCGT
CGGGCGATGCTCCACCGCTGGTGGACGACTCGGAGGATCTGTCGCACATGGAGGAGGTCGATTAA
>(AGA)HSP90A3P
MPEPQEGETFAFQAEIAQLMSLIINTFYSNKEIFLRELISNSSDALDKIRYESLTDPSKLESGKELFIKIIPNKEAG
TLTLIDTGIGMTKADLVNNLGTIAKSGTKAFMEALQAGADISMIGQFGVGFYSAYLVADKVVVTSKNNDDEQYVWES
SAGGSFTVRPDSGEPLGRGTKIVLHIKGGPAGVPGGEQDQADREQALAEDKAKFENLCKVMKSVLESKVEKVMVSNR
LVDSPCCIVTSQYGWSANMERIMKAQALRDSSAMGYMAGKKHLEINPDHAIIETLRQRAEADKNDKAVKDLVILLFE
TALLSSGFSLDEPGTHASRIYRMIKLGLGIDEDEPMTTEDSSSGAAAAAPASGDAPPLVDDSEDLSHMEEVD
>(AGA)HSP90B1N
ATGAAGTACTTGCTACTTCTAGTAGTGGGTGTGTTCCTGGTGTCAGGAATTCACCAAGTGCGTGCGGATGATGACGA
TGACACACTGCCAATGGTGGACAATGATCTCGGCGCCTCGAAGGAAGGTTCGCGCACCGACGCGGAGGCGGTCAAGC
GCGAAGAGGAAGCCATCAAGCTGGACGGTCTGAACGTGGCCCAGATCAAGGAGCTGCGGGAAAAGTCGGAAAAGTTC
ACGTTCCAGGCGGAGGTGAACCGCATGATGAAGCTCATCATCAACTCGCTGTACCGCAACAAGGAGATTTTCCTGCG
TGAGCTGATCTCGAACGCTTCGGACGCGCTGGACAAGATCCGCCTGCTGTCCCTGACCGACCCGTCCGTGCTGGACA
GCAACCGCAACCTGGAGGTGAAGATCAAGGCCGACAAGGAGGGCAAGGTGCTGCACATCATCGACACCGGCATCGGT
ATGACGAAGCAGGATCTGGTGAACAATCTCGGTACGATCGCGAAGTCGGGCACGGCCGACTTCCTGTCGAAGATGCA
GGACAAGGAGAAGGCCGACGGGCAGGACGTGAACGACATGATTGGCCAGTTCGGTGTCGGCTTCTACTCGGCGTTCC
TGGTGGCCGACCGGGTCGTGGTGACGACGAAGCACAACGACGACAAGCAGTACATCTGGGAGTCGGATGCGGCCAGC
TTCAGCATTGTGGAGGACCCGCGCGGCAACACGCTGGAGCGCGGTTCGCAGGTCTCGCTGCACCTGAAGGAGGAAGC
GCTCGACTTCCTGGAGGACGATACGGTGAAGCAGCTGATCAAGAAGTACTCGCAGTTCATCAACTTCCCGATCTACA
TGTGGACGAGCAAGGAGGTGGAGGAGGAGGTGGCCGTCGAGGAGGAGGCGACCGAGAAGCCGGCCAAGAGCGAGGAC
AGCACGGACGAGGAGGAGGAGGACGTGAAGGTGGAGGAGGAGGAGGCGACCGACAGCGACAAGCCGAAGACGAAGAA
GGTGAAGAAGACGGTCTGGAACTGGGAGATCATGAACGACAGCAAACCGATCTGGACGCGCAAGGTGAGCGACGTGA
CCGACGAGGAGTACACCGAGTTCTACAAGAGCCTGACGAAGGACACGTCCGAACCGCTGACGCACACGCACTTCATT
GCCGAGGGTGAGGTCACGTTCAAGTCGCTGCTGTTCGTGCCGAAGGTGCAGCCGTCGGAGAGCTTCAACAAATACGG
CACCAAGGCGGACAACATCAAGCTGTACGTGCGGCGCGTGTTCATTACGGACGAGTTCAACGACATGATGCCAAACT
ATCTGAGCTTCATCCGGGGCGTGGTCGATTCGGACGATCTGCCGCTGAACGTGTCGCGCGAAACGCTCCAGCAGCAC
AAGCTGATCAAGGTGATCAAGAAGAAGCTCGTGCGCAAGGCGCTGGACATGATCAAGAAGATCGACAAGGAGCAGTA
CGAGCAGTTCTGGAAGGAGTACTCCACCAACATCAAGCTGGGCATCATGGAGGACCCGAGCAATCGGTCGCGCCTGG
CCAAGCTGCTCCGCTTCCAGTCGTCCTCGACGAAGAACAAGGAGTACACCAGCCTGTCCGACTACGTGGCGCGCATG
AAGCCGAAGCAGGACAACATCTACTTCATCGCCGGCCCGAACCGTGCCGAGATCGAGAAGTCGCCGTTCGTCGAGCG
TCTGCTGTCCCGCGGCTACGAAGTCCTGTTCCTGGTGGAGGCCGTCGACGAGTACAGCATCTCCGCGCTGCCCGAGT
TCGACGGCAAGCGCTTCCAGAACGTCGCCAAGGAGGGCTTCACGTTGAACGAGTCGGAAGAGTCGAAGGCCCGTTTC
GAGGAGCTGAAGACGGAGTACGAACCCCTGCTGAAGTGGTTGAACGATGTCGCACTGAAGGACAAAATCGCCAAAGC
CCAGCTGTCCGAGCGACTGTCCAACTCGCCCTGCGCTCTCGTCGCGTCGATGTTCGGCTGGACCGGCAACATGGAGC
GGCTGGCGCTCGCCAACGCTCACCAGAAGACGGACGACCCGCAGCGCCACTACTACCTGAACCAGAAGAAAACGCTC
GAAATCAACCCGCGCCATCCGCTGATGCGCGAGCTGTTGCGCCGCGTCGAGGTCGATTCGGACGATATCGTGGCGAA
GGATATGGCGGTGCTGATGTTCAACACGGCCACGCTGCGGTCCGGCTTCCAGCTGCCGGAGACGGCCGACTTTGCCG
ACAGTGTCGAGCGGATGATGCGCCAAACGTTGGGCGTTTCGCTGGACGAGCAGCCCGAGCAGGAGGAGTTCGTGGAC
GAACCGGCGGCTGGCGGTGAGGAGGGCGGCGCTGCCGCCGACGAGGACGAAGAATCCATCGATGCCGATGGTGGTGA
CCACGACGAGCTGTAA
>(AGA)HSP90B1P
MKYLLLLVVGVFLVSGIHQVRADDDDDTLPMVDNDLGASKEGSRTDAEAVKREEEAIKLDGLNVAQIKELREKSEKF
TFQAEVNRMMKLIINSLYRNKEIFLRELISNASDALDKIRLLSLTDPSVLDSNRNLEVKIKADKEGKVLHIIDTGIG
MTKQDLVNNLGTIAKSGTADFLSKMQDKEKADGQDVNDMIGQFGVGFYSAFLVADRVVVTTKHNDDKQYIWESDAAS
FSIVEDPRGNTLERGSQVSLHLKEEALDFLEDDTVKQLIKKYSQFINFPIYMWTSKEVEEEVAVEEEATEKPAKSED
STDEEEEDVKVEEEEATDSDKPKTKKVKKTVWNWEIMNDSKPIWTRKVSDVTDEEYTEFYKSLTKDTSEPLTHTHFI
AEGEVTFKSLLFVPKVQPSESFNKYGTKADNIKLYVRRVFITDEFNDMMPNYLSFIRGVVDSDDLPLNVSRETLQQH
KLIKVIKKKLVRKALDMIKKIDKEQYEQFWKEYSTNIKLGIMEDPSNRSRLAKLLRFQSSSTKNKEYTSLSDYVARM
KPKQDNIYFIAGPNRAEIEKSPFVERLLSRGYEVLFLVEAVDEYSISALPEFDGKRFQNVAKEGFTLNESEESKARF
EELKTEYEPLLKWLNDVALKDKIAKAQLSERLSNSPCALVASMFGWTGNMERLALANAHQKTDDPQRHYYLNQKKTL
EINPRHPLMRELLRRVEVDSDDIVAKDMAVLMFNTATLRSGFQLPETADFADSVERMMRQTLGVSLDEQPEQEEFVD
EPAAGGEEGGAAADEDEESIDADGGDHDEL
>(AGA)TRAP1N
ATGAGCGTTGTTAACGGATTACTGCGCGTCGGCCGGCTGATTAGGCCGTCCACAACGCTTGCCAAATGCTTGCTGCC
GGCCACTGGTGCTCCTCAATCGGCATGGGCCACCATCGGACAAGTGCATCAACAGCCGCAGCGATCGTGGCTTTACG

CCCACCGGACACTGAGCACCAAGGTGGCGGAAGAAGGATATCACACGATTATACGCGACCAAGAGAAGGCAGTTGGG
ACGAGCGACAAGCACGAGTTCCAGGCGGAAACGCGCATGCTGCTCGACATCGTGGCCCGTTCGCTCTACTCCGACAA
GGAGGTGTTTGTCCGGGAGCTCGTATCGAACGCTAGCGATGCGCTGGAAAAGTTCCGCTTTCTCGTGCAAACATCGA
CCGGCGCGGCCAGCGATGGTGAAGCGGGTGAGTTCGCGGAAGCGGACCGCTCGCTCGAAATACACATCGGCACGAAC
AAGCAGGACCGTCAGTTAACGATCCAGGATACCGGCATTGGGATGACGCGGGACGAGCTGGTGGCCAATCTCGGCAC
CATTGCCCGGTCCGGCTCGAAACAGTTCATGGAGCAGCTGAAGGAGAGTGGGCGCGGAACGCAAGAAAACGTGCAAA
ACATTATCGGCCAGTTTGGCGTTGGGTTTTACTCGGCGTTCATGGTAGCGGATCGGGTGGACGTGTACACTCGCTCG
TCCCGGGCCGGTGCGCCCGGCCTCAAGTGGTCGTCCGATGGTTCGGGTACGTTCGAAATTCAGGAGGCGGAGAATGT
GGCCATCGGCACTAAGATTGTGATCCATCTGAAGGCGGACTGTCGGGAGTTTGCGGACGAGGACCGGATCCGGGAGG
TGATCCGGCGCTACAGCAACTTCGTGGGCAGTCCCATCTTCCTGAACGGCAAGCAGGCGAACCAGATCCAGCCGATC
TGGCTGATGGAACCGAAGCAGGTGACGCCCGAACAGCACAACGAGTTCTACCGGTTCGTGGGCAACACGTTCGACAC
GCCCCGCTTTACGCTGCACTACAAAACGGACGTTCCGCTCAGCATTCGGGCGCTGCTGTACTTCCCCGAAGGTAAGC
CGGGCCTGTTCGAGATGTCGCGCGATGCGGACGGCGGTGTGGCCCTGTACACGCGCAAGGTGCTGATCCAATCCAAG
ACGGAGAACCTGCTGCCCAAGTGGCTGCGCTTCCTCAAGGGCGTCGTCGATTCGGAAGACATTCCGCTCAATCTGAG
CCGCGAGCTGCTGCAAAACAGTGCGCTCATTCGGAAGCTGCGCACGGCGCTAACGAACCGCACGCTGCGCTTCCTGC
ACGACCGCTCGCAAAAGGAACCGGAAAGCTACGACAAGTTCTACAAAGACTACGGGCTCTTCCTGAAGGAAGGCATC
GTGACCAGCCAGGAGCAGCAGGAAAAGGAAGAGATCGCCAAGCTGCTGCGGTTCGAAACGAGCAAGGAACCGAACCG
GACGGTATCGCTGCCCGAGTACTGCCAGCGGCAGGCGGAAGGCCAGAAGGATATATACTATCTGGCCGCGCCGAATC
GTACCCTGGCGGAAGCTTCCCCGTACTATGAGTCGCTGAAGAAGCGCGGCATCGAGGTGCTGTTCTGTTACGAAGCG
TACGATGAGCTGGTGCTGATGCAGCTCGGCATGTATTTGGGCAAGAATTTAGTATCGGTGGAGAAGGAGATGCGCCG
TTCCGATGCGTCGACCGAAGGGAAGGATGCGGACGGGCTGATCGAAGGTTCGCTACTGAAAACGCAAATCGATGAGC
TGCTGCCGTGGCTGAAGGACAAACTAACCGGCAAGGTGTCGAACGTGAAGACGACCGGCAAGCTCGATACGCATCCG
TGTGTGGTGACGGTGGAGGAGATGGCCGCTGCCCGGCACTTTATCAAAACGCAGAGCCACAACATTAGCGAAGAGAA
CCGTTACGCACTGTTGCAACCGCAGTTTGAAATCAATCCCAAACATCCCATCATTAAGAAGCTACACAAACTAACGA
GCAGCGATCCGGAACTGGCCGAACTGTTGGCCAAGCAGCTGTTCTCGAACGCGATGGTGGGCGCCGGTTTGGTGGAT
GATCCGCGCATGCTGCTGACGAGCATGAACGATCTGCTGCAAAAGGTACTTGATAAGCATTAG
>(AGA)TRAP1P
MSVVNGLLRVGRLIRPSTTLAKCLLPATGAPQSAWATIGQVHQQPQRSWLYAHRTLSTKVAEEGYHTIIRDQEKAVG
TSDKHEFQAETRMLLDIVARSLYSDKEVFVRELVSNASDALEKFRFLVQTSTGAASDGEAGEFAEADRSLEIHIGTN
KQDRQLTIQDTGIGMTRDELVANLGTIARSGSKQFMEQLKESGRGTQENVQNIIGQFGVGFYSAFMVADRVDVYTRS
SRAGAPGLKWSSDGSGTFEIQEAENVAIGTKIVIHLKADCREFADEDRIREVIRRYSNFVGSPIFLNGKQANQIQPI
WLMEPKQVTPEQHNEFYRFVGNTFDTPRFTLHYKTDVPLSIRALLYFPEGKPGLFEMSRDADGGVALYTRKVLIQSK
TENLLPKWLRFLKGVVDSEDIPLNLSRELLQNSALIRKLRTALTNRTLRFLHDRSQKEPESYDKFYKDYGLFLKEGI
VTSQEQQEKEEIAKLLRFETSKEPNRTVSLPEYCQRQAEGQKDIYYLAAPNRTLAEASPYYESLKKRGIEVLFCYEA
YDELVLMQLGMYLGKNLVSVEKEMRRSDASTEGKDADGLIEGSLLKTQIDELLPWLKDKLTGKVSNVKTTGKLDTHP
CVVTVEEMAAARHFIKTQSHNISEENRYALLQPQFEINPKHPIIKKLHKLTSSDPELAELLAKQLFSNAMVGAGLVD
DPRMLLTSMNDLLQKVLDKH
>(DRE)HSP90AA2N
ATGCCTGAGGCTCACGAGCAGCAGATGATGGAGGATGAGGAGGTGGAGACGTTTGCGTTTCAGGCTGAGATCGCTCA
GCTCATGTCTCTGATCATCAACACCTTCTACTCCAACAAGGAGATCTTCCTCCGAGAGCTCATCTCCAACTCCTCTG
ATGCTTTGGACAAAATCCGCTATGAAAGTCTCACAGACCCAAGCAAGCTGGACTCAGGAAAAGACCTTAAAATCGAA
ATCATTCCCAACAAAGAAGAGCGCACGCTGACCATCATCGACACCGGCATCGGCATGACCAAAGCCGACCTGATCAA
CAATCTGGGCACCATCGCTAAATCCGGCACAAAGGCCTTCATGGAGGCTCTGCAGGCCGGAGCGGACATTTCTATGA
TCGGTCAGTTCGGTGTGGGCTTTTATTCTGCGTATCTGGTGGCCGAGAAAGTGACGGTCATCACCAAACATCTCGAT
GATGAGCAGTACGCGTGGGAATCGTCTGCTGGCGGATCGTTCACTGTCAAAGTGGATAACTCTGAGCCAATCGGCCG
TGGCACAAAAGTGATTCTTCATCTGAAGGAGGATCAGACAGAGTACATCGAAGAGCGGCGGATCAAAGAGATCGTCA
AGAAACACTCGCAGTTTATTGGATACCCTATCACACTCTTTGTGGAGAAGGAGCGCGATAAGGAGGTGAGCGATGAT
GAGGCGGAAGAGGAGAAAGAGAAGGAGAAAGAAGAAGAAGAAGAGGGCGAGAAAGACGAAGACAAGCCTGAGATTGA
AGACGTAGGCTCAGATGAGGACGACCACGATCATGGCGATAAGTGTGGCGACAAGAAGAAGAAAAAGAAGAAGATCA
AGGAGAAATACATCGATCAGGAGGAGCTGAACAAAACCAAACCGCTGTGGACCCGCAACCCTGATGACATCACCAAC
GAAGAGTACGGAGAGTTTTACAAGAGCCTGACCAACGACTGGGAGGATCATCTGGCTGTTAAGCACTTTTCAGTGGA
AGGTCAGCTGGAATTTCGCGCGCTGCTTTTTGTCCCTCGTCGAGCTCCATTCGATCTGTTTGAGAACAAGAAAAAGA
AGAATAACATCAAACTGTACGTGCGCAGGGTGTTCATCATGGACAACTGTGACGAGCTCATACCAGAATATCTCAAC
TTTATTAAGGGTGTGGTGGACTCTGAGGATCTGCCTCTGAACATCTCCAGAGAAATGCTTCAACAGAGCAAAATCCT
GAAGGTGATCCGCAAAAACCTGGTCAAGAAGTGTCTGGAGCTCTTCACCGAACTGGCAGAGGATAAAGACAACTATA
AGAAATACTACGAGCAGTTCTCCAAAAACATCAAGCTGGGCATCCATGAAGACTCGCAGAACAGAAAGAAACTGTCA
GAGCTGCTGCGCTACTACACATCCGCTTCAGGAGACGAGATGGTGTCGCTCAAAGATTACGTCACACGCATGAAGGA

CACGCAGAAGCACATCTACTACATCACCGGTGAGACCAAAGATCAGGTGGCGAACTCAGCGTTTGTGGAGCGCCTCC
GTAAAGCAGGTCTGGAGGTGATCTACATGATCGAGCCCATCGACGAATACTGTGTTCAGCAGCTGAAGGAGTTCGAA
GGCAAGAATCTGGTGTCGGTCACTAAAGAAGGCCTGGAGCTGCCCGAGGATGAGGAGGAGAAGAAGAAGCAGGAGGA
GAAGAAGAGCAAGTTTGAAAACCTCTGCAAGATCATGAAGGACATCCTGGAGAAGAAAGTGGAGAAGGTCACCGTCT
CCAACCGTCTGGTGTCTTCGCCTTGCTGTATCGTCACCAGCACATACGGCTGGACGGCGAACATGGAGAGGATCATG
AAGGCTCAGGCCCTCAGAGATAACTCCACTATGGGTTACATGGCTGCCAAAAAGCACCTGGAGATCAACCCTGACCA
CCCCATCGTGGAGACTCTCCGGCAGAAAGCGGAAGCTGACAAAAACGACAAATCTGTGAAGGATCTGGTGATCCTGC
TGTTCGAGACGGCGCTCTTATCCTCAGGCTTCACACTGGACGACCCGCAGACACACTCCAACCGCATCTACAGGATG
ATCAAACTGGGCTTAGGCATTGATGAGGACGACCTGTCGGCAGAGGAGCCCAGTTCTGCCCCGATAGAGGAGATGCC
GCCGCTGGAAGGAGACGACGACACATCCCGCATGGAGGAGGTGGACTGA
>(DRE)HSP90AA2P
MPEAHEQQMMEDEEVETFAFQAEIAQLMSLIINTFYSNKEIFLRELISNSSDALDKIRYESLTDPSKLDSGKDLKIE
IIPNKEERTLTIIDTGIGMTKADLINNLGTIAKSGTKAFMEALQAGADISMIGQFGVGFYSAYLVAEKVTVITKHLD
DEQYAWESSAGGSFTVKVDNSEPIGRGTKVILHLKEDQTEYIEERRIKEIVKKHSQFIGYPITLFVEKERDKEVSDD
EAEEEKEKEKEEEEEGEKDEDKPEIEDVGSDEDDHDHGDKCGDKKKKKKKIKEKYIDQEELNKTKPLWTRNPDDITN
EEYGEFYKSLTNDWEDHLAVKHFSVEGQLEFRALLFVPRRAPFDLFENKKKKNNIKLYVRRVFIMDNCDELIPEYLN
FIKGVVDSEDLPLNISREMLQQSKILKVIRKNLVKKCLELFTELAEDKDNYKKYYEQFSKNIKLGIHEDSQNRKKLS
ELLRYYTSASGDEMVSLKDYVTRMKDTQKHIYYITGETKDQVANSAFVERLRKAGLEVIYMIEPIDEYCVQQLKEFE
GKNLVSVTKEGLELPEDEEEKKKQEEKKSKFENLCKIMKDILEKKVEKVTVSNRLVSSPCCIVTSTYGWTANMERIM
KAQALRDNSTMGYMAAKKHLEINPDHPIVETLRQKAEADKNDKSVKDLVILLFETALLSSGFTLDDPQTHSNRIYRM
IKLGLGIDEDDLSAEEPSSAPIEEMPPLEGDDDTSRMEEVD
>(DRE)HSP90AA3N
ATGCCTGAGAAGTCGGCCCAGCCCGTGATGGAGGAGGAGGTGGAGACGTTTGCGTTTCAGGCTGAGATCGCTCAGCT
CATGTCTCTGATCATCAACACCTTCTACTCCAACAAGGAGATCTTCCTCCGAGAGCTCATCTCCAACTCCTCTGATG
CATTGGACAAGATCAGATATGAGAGCTTGACAGATCCGAGCAAACTGGATTCAAGGGCCCTGAAGATTGAACTGATC
CCAGACCAGAAAGAGCGCACGCTGACCATCATCGACACCGGCATCGGCATGACCAAAGCTGACCTGATCAACAATCT
GGGCACCATCGCTAAATCTGGCACAAAGGCCTTCATGGAGGCTCTGCAGGCCGGAGCGGACATTTCTATGATCGGTC
AGTTCGGTGTGGGCTTTTATTCTGCGTATCTGGTGGCTGAGAAAGTGACGGTCATCACCAAACACAACGATGATGAG
CAGTACATTTGGGAATCTGCAGCTGGCGGATCGTTCACTGTCAAGCCAGACTTCGGTGAATCAATTGGACGTGGTAC
CAAAGTCATTCTCCACCTTAAAGAGGATCAGTCTGAATATGTGGAGGAAAAGCGCATTAAGGAAGTGGTGAAGAAGC
ACTCTCAGTTCATCGGTTACCCAATTACGCTTTACATTGAGAAACAGAGAGAAAAAGAGGTGGATCTTGAGGAAGGC
GAGAAACAGGAGGAGGAGGAGGTTGCAGCAGGCGAAGACAAAGACAAACCCAAAATTGAAGATCTGGGAGCTGATGA
AGATGAAGACTCTAAGGATGGCAAGAATAAGAGGAAGAAGAAGGTGAAGGAAAAGTACATTGATGCCCAGGAGCTGA
ATAAGACCAAGCCGATCTGGACCCGTAACCCCGATGACATCACCAATGAGGAGTACGGCGAGTTTTACAAGAGTTTA
AGCAATGACTGGGAGGATCACTTGGCTGTCAAGCATTTCTCAGTTGAGGGCCAACTGGAGTTTCGCGCTCTACTTTT
TGTACCCAGAAGAGCCGCTTTTGACCTTTTTGAGAACAAGAAGAAGAGAAACAACATTAAGTTATACGTGCGCAGGG
TTTTCATCATGGACAATTGCGAGGAACTCATTCCAGAATATCTCAACTTTATTAAGGGTGTGGTGGACTCTGAGGAT
CTGCCTCTGAACATCTCCAGAGAAATGCTTCAACAGAGCAAAATCCTGAAGGTGATCCGCAAAAACCTGGTCAAGAA
GTGTCTCGATCTCTTCACCGAACTGGCAGAGGATAAAGACAACTATAAGAAATACTACGAGCAGTTCTCCAAAAACA
TCAAGCTTGGCATCCACGAGGACTCTCAGAATCGCAAGAAACTTTCTGATCTTTTGCGCTACTACACTTCAGCTTCA
GGAGATGAGATGGTGTCACTTAAAGACTATGTGTCTCGCATGAAGGACACACAGAAGCACATCTACTACATCACCGG
TGAGACCAAAGATCAGGTGGCGAACTCAGCGTTTGTGGAGCGCCTCCGTAAAGCTGGTCTGGAGGTGATCTACATGA
TCGAGCCCATCGATGAATACTGTGTTCAGCAGCTGAAGGAATATGATGGCAAGAATCTGGTGTCGGTCACTAAAGAA
GGCCTGGAGCTGCCCGAGGATGAAGAGGAGAAGAAGAAGCAGGATGAGCTGAAGGCCAAATATGAGAACTTGTGCAA
GATCATGAAGGATATCCTCGACAAAAAGATTGAGAAGGTCACAGTCTCCGACCGTCTGGTGTCTTCGCCCTGCTGTA
TCGTCACCAGCACATACGGCTGGACGGCGAACATGGAGAGGATCATGAAGTCTCAGGCTCTGAGGGATAACTCTACC
ATGGGCTACATGACCGCTAAAAGGCACCTAGAAATAAACCCAGCACACCCCATCGTGGAGACCCTCAGAGAGAAAGC
GGAAGCCGACAAAAACGACAAGGCGGTGAAGGATCTGGTGATCCTGCTGTTCGAGACGGCGCTCTTATCCTCAGGCT
TCACACTGGACGACCCACAGACTCATGCAAACCGCATCTACAGGATGATCAAACTCGGACTTGAGAAGGAGAAAGAA
GAAGAAGAAGAGGGCGAGAAAGACGAAGACAAGCCTGAGATTGAAGACGTAGGCTCAGATGAGGACGACCACGATCA
TGGCGATAAGTGTGGCGACAAGAAGAAGAAAAAGAAGAAGATCAAGGAGAAATACATCGATCAGGAGGAGCTGAACA
AAACCAAACCGCTGTGGACCCGCAACCCTGATGACATCACCAACGAAGAGTACGGAGAGTTTTACAAGAGCCTGACC
AACGACTGGGAGGATCATCTGGCTGTTAAGCACTTTTCAGTGGAAGGTCAGCTGGAATTTCGCGCGCTGCTTTTTGT
CCCTCGTCGAGCTCCATTCGATCTGTTTGAGAACAAGAAAAAGAAGAATAACATCAAACTGTACGTGCGCAGGGTGT
TCATCATGGACAACTGTGACGAGCTCATACCAGAATATCTCAGTATTAAATGA
>(DRE)HSP90AA3P
MPEKSAQPVMEEEVETFAFQAEIAQLMSLIINTFYSNKEIFLRELISNSSDALDKIRYESLTDPSKLDSRALKIELI

PDQKERTLTIIDTGIGMTKADLINNLGTIAKSGTKAFMEALQAGADISMIGQFGVGFYSAYLVAEKVTVITKHNDDE
QYIWESAAGGSFTVKPDFGESIGRGTKVILHLKEDQSEYVEEKRIKEVVKKHSQFIGYPITLYIEKQREKEVDLEEG
EKQEEEEVAAGEDKDKPKIEDLGADEDEDSKDGKNKRKKKVKEKYIDAQELNKTKPIWTRNPDDITNEEYGEFYKSL
SNDWEDHLAVKHFSVEGQLEFRALLFVPRRAAFDLFENKKKRNNIKLYVRRVFIMDNCEELIPEYLNFIKGVVDSED
LPLNISREMLQQSKILKVIRKNLVKKCLDLFTELAEDKDNYKKYYEQFSKNIKLGIHEDSQNRKKLSDLLRYYTSAS
GDEMVSLKDYVSRMKDTQKHIYYITGETKDQVANSAFVERLRKAGLEVIYMIEPIDEYCVQQLKEYDGKNLVSVTKE
GLELPEDEEEKKKQDELKAKYENLCKIMKDILDKKIEKVTVSDRLVSSPCCIVTSTYGWTANMERIMKSQALRDNST
MGYMTAKRHLEINPAHPIVETLREKAEADKNDKAVKDLVILLFETALLSSGFTLDDPQTHANRIYRMIKLGLEKEKE
EEEEGEKDEDKPEIEDVGSDEDDHDHGDKCGDKKKKKKKIKEKYIDQEELNKTKPLWTRNPDDITNEEYGEFYKSLT
NDWEDHLAVKHFSVEGQLEFRALLFVPRRAPFDLFENKKKKNNIKLYVRRVFIMDNCDELIPEYLSIK
>(DRE)HSP90AA4N
ATGCCTGAGAAGTCGGCCCAGCCCGTGATGGAGGAGGAGGTGGAGACGTTTGCGTTTCAGGCTGAGATCGCTCAGCT
CATGTCTCTGATCATCAACACCTTCTACTCCAACAAGGAGATCTTCCTCCGAGAGCTCATCTCCAACTCCTCTGATG
CATTGGACAAGATCAGATATGAGAGCTTGACAGATCCGAGCAAACTGGATTCATGCAAGCTGAAGATTGAACTGATC
CCAGACCAGAAAGAGCGCACGCTGACCATCATCGACACCGGCATCGGCATGACCAAAGCTGACCTGATCAACAATCT
GGGCACCATCGCTAAATCTGGCACAAAGGCCTTCATGGAGGCTCTGCAGGCCGGAGCGGACATTTCTATGATCGGTC
AGTTCGGTGTGGGCTTTTATTCTGCGTATCTGGTGGCTGAGAAAGTGACGGTCATCACCAAACACAACGATGATGAG
CAGTACATTTGGGAATCTGCAGCTGGCGGATCGTTCACTGTCAAGCCAGACTTCGGTGAATCAATTGGACGTGGTAC
CAAAGTCATTCTCCACCTTAAAGAGGATCAGTCTGAATATGTGGAGGAAAAGCGCATTAAGGAAGTGGTGAAGAAGC
ACTCTCAGTTCATCGGTTACCCAATTACGCTTTACGTGGAGAAGGAGCGCGATAAGGAGGTGAGCGATGATGAGGCG
GAAGAGGAGAAAGAGAAGGAGAAAGAAGAAGAAGAAGAGGGCGAGAAAGACGAAGACAAGCCTGAGATTGAAGACGT
AGGCTCAGATGAGGACGACCACGATCATGGCGATAAGTGTGGCGACAAGAAGAAGAAAAAGAAGAAGATCAAGGAGA
AATACATCGATCAGGAGGAGCTGAACAAAACCAAACCGCTGTGGACCCGCAACCCTGATGACATCACCAACGAAGAG
TACGGAGAGTTTTACAAGAGCCTGACCAACGACTGGGAGGATCATCTGGCTGTTAAGCACTTTTCAGTGGAAGGTCA
GCTGGAATTTCGCGCGCTGCTTTTTGTCCCTCGTCGAGCTCCATTCGATCTGTTTGAGAACAAGAAAAAGAAGAATA
ACATCAAACTGTACGTGCGCAGGGTGTTCATCATGGACAACTGTGACGAGCTCATACCAGAATATCTCAGTAAGTGT
CTGATCACAAACACTAATATCAGGTTTAATTGGTTGATGTTCACATTTTATGCCTTAAAAAGTCTTAAATACACAGA
AAAATTGTGTTGTAGGTATTAA
>(DRE)HSP90AA4P
MPEKSAQPVMEEEVETFAFQAEIAQLMSLIINTFYSNKEIFLRELISNSSDALDKIRYESLTDPSKLDSCKLKIELI
PDQKERTLTIIDTGIGMTKADLINNLGTIAKSGTKAFMEALQAGADISMIGQFGVGFYSAYLVAEKVTVITKHNDDE
QYIWESAAGGSFTVKPDFGESIGRGTKVILHLKEDQSEYVEEKRIKEVVKKHSQFIGYPITLYVEKERDKEVSDDEA
EEEKEKEKEEEEEGEKDEDKPEIEDVGSDEDDHDHGDKCGDKKKKKKKIKEKYIDQEELNKTKPLWTRNPDDITNEE
YGEFYKSLTNDWEDHLAVKHFSVEGQLEFRALLFVPRRAPFDLFENKKKKNNIKLYVRRVFIMDNCDELIPEYLSKC
LITNTNIRFNWLMFTFYALKSLKYTEKLCCRY
>(DRE)HSP90B2N
ATGAGGCGACTGTGGATTATCGGTCTCCTCTGTGCACTTTTGGCGTTCGCATCTGTAAAAGCTGATGATGATGATGT
TGACATTGATGGCACAGTAGAAGAGGACCTTGGGAAGAGCAGAGACGGATCCCGCACCGATGACGAGGTTGTTCAGA
GGGAGGAGGAGGCCATTCAGCTAGATGGTTTAAACACCTCACAATTAAAGGAAATTCGTGATAAAGCAGAAAAGCAT
GCGTTCCAGGCAGAAGTGAATCGAATGATGAAACTGATCATCAATTCTCTTTATAAGAACAAAGAGATCTTCCTGAG
AGAGCTGATCTCTAATGCTTCCGATGCCTTGGATAAGATCCGGCTGCTGTCTCTGACCAATGAAGATGCCCTTGCCG
GAAATGAAGAGCTCACTATTAAAATTAAGTCTGACAAAGAGAAGAATATGCTTCACATCACTGACACTGGTATTGGC
ATGACCAAAGAAGAGCTGGTGAAGAACCTTGGTACCATCGCCAAATCTGGAACCAGCGAGTTCCTGAACAAAATGAC
AGAGGTGCAGGACGACAGTCAGTCCACCTCTGAGCTGATCGGTCAGTTCGGTGTGGGCTTCTACTCCGCTTTCCTTG
TGGCCGATAAGGTCATTGTCACTTCCAAGCACAACAACGACACCCAGCACATGTGGGAATCCGATTCCAATCAGTTC
TCTGTCATCGAGGACCCACGTGGAGACACTCTAGGCAGAGGCACCACCATCACGTTGGTGATGAAAGAGGAAGCTTC
TGACTACCTTGAGCTGGAGACCATTAAGAACCTGGTGAAGAAATACTCCCAATTCATCAACTTCCCCATCTACGTAT
GGAGCAGCAAGACCGAGACCGTGGAAGAGCCTATTGAGGATGAGGCTGAGGCAGAGAAGGAAGAAGCCACTGAAGAT
GAAGCTGAAGTTGAGGAAGAGGAGGAAGACAAGGACAAACCAAAGACTAAGAAGGTGGAGAAGACTGTGTGGGATTG
GGAGCTGATGAATGACATTAAACCCATCTGGCAGAGACCTGCAAAGGAAGTGGAGGAAGATGAATATACAGCCTTCT
ATAAAACCTTCTCCAGGGACACTGATGAGCCACTGTCACACATTCACTTCACCGCTGAAGGCGAAGTCACTTTCAAG
TCCATCCTCTTTGTTCCTGCATCTGCACCCAGAGGTCTTTTCGACGAGTATGGCACCAAGAAGAATGACTTCATTAA
GCTGTTCGTGCGTAGAGTCTTCATCACTGATGACTTCCATGACATGATGCCCAAGTACCTCAACTTCATCAAGGGTG
TTGTGGACTCTGACGATCTGCCTCTGAACGTGTCCAGAGAGACTCTGCAGCAACACAAACTGCTCAAGGTTATCCGT
AAAAAGCTGGTGCGCAAGACCCTGGATATGATCAAGAAGATCGCTGAGGAGCAGTACAACGATAAGTTCTGGAAAGA
GTTTGGCACCAACATTAAGCTGGGTGTGATTGAGGATCACTCCAACAGAACCCGCCTGGCCAAACTGCTCCGCTTCC
AGACCTCCCACAGCGACACCGTGCTGTCCAGTCTGGAGCAGTATGTGGAGAGGATGAAGGAGAAGCAGGACAAGATC

TACTTCATGGCTGGAACAAGCAGGAAGGAGGCTGAATCATCTCCATTTGTGGAGAAGCTTCTTAAGAAAGGATATGA
AGTGGTCTATCTGACTGAACCAGTGGATGAGTACTGCATTCAGGCCCTTCCTGAGTTTGATGGCAAACGCTTCCAGA
ACGTGGCGAAGGAGGGTGTGAAGTTCGACGAGAGCGATAAGGCCAAGGAAAAGAGGGAAGCTTTGGAGAAAGAGTTT
GAGCCCCTTACTACCTGGATGAAAGATAAGGCCCTGAAGGAACAAATCGAGAAGGCTGTTTTGTCCCAAAGGTTGAC
AAACTCTCCATGTGCTCTGGTTGCCAGTCAGTATGGATGGTCTGGAAACATGGAACGTATCATGAAAGCTCAGGCTT
ACCAGACAGGAAAAGACATTTCCACAAATTATTACGCAAGCCAAAAGAAGACATTAGAAATCAACCCCAAACATCCA
CTCATCAAAGAGATGCTAAGGAGAGTCAACGAAGATGCTGAGGATAAGACTGCCGCAGATTTAGCCGTAGTGCTGTT
TGAGACCGCCACACTGCGATCAGGATACCAGCTCCAAGACACCAAAGCCTACGGAGAAAGAATAGAGCGCATGCTGC
GGCTCAGCATGAATGTAGACCTTGACGCTCAGGTAGAGGAAGAGCCAGAGGAGGAACCAGAAGAACAGACAGAGGAA
GCTGAGGATGAGGAGGAGGTCCAGGCAGATGAAGCAGAAGAGGAATCAGAGGCCACATCCAAAGATGAGCTGTAA
>(DRE)HSP90B2P
MRRLWIIGLLCALLAFASVKADDDDVDIDGTVEEDLGKSRDGSRTDDEVVQREEEAIQLDGLNTSQLKEIRDKAEKH
AFQAEVNRMMKLIINSLYKNKEIFLRELISNASDALDKIRLLSLTNEDALAGNEELTIKIKSDKEKNMLHITDTGIG
MTKEELVKNLGTIAKSGTSEFLNKMTEVQDDSQSTSELIGQFGVGFYSAFLVADKVIVTSKHNNDTQHMWESDSNQF
SVIEDPRGDTLGRGTTITLVMKEEASDYLELETIKNLVKKYSQFINFPIYVWSSKTETVEEPIEDEAEAEKEEATED
EAEVEEEEEDKDKPKTKKVEKTVWDWELMNDIKPIWQRPAKEVEEDEYTAFYKTFSRDTDEPLSHIHFTAEGEVTFK
SILFVPASAPRGLFDEYGTKKNDFIKLFVRRVFITDDFHDMMPKYLNFIKGVVDSDDLPLNVSRETLQQHKLLKVIR
KKLVRKTLDMIKKIAEEQYNDKFWKEFGTNIKLGVIEDHSNRTRLAKLLRFQTSHSDTVLSSLEQYVERMKEKQDKI
YFMAGTSRKEAESSPFVEKLLKKGYEVVYLTEPVDEYCIQALPEFDGKRFQNVAKEGVKFDESDKAKEKREALEKEF
EPLTTWMKDKALKEQIEKAVLSQRLTNSPCALVASQYGWSGNMERIMKAQAYQTGKDISTNYYASQKKTLEINPKHP
LIKEMLRRVNEDAEDKTAADLAVVLFETATLRSGYQLQDTKAYGERIERMLRLSMNVDLDAQVEEEPEEEPEEQTEE
AEDEEEVQADEAEEESEATSKDEL
>(DRE)TRAP1N
ATGATCACGGCTGGAGGAGACACAGCACCAATGGAGATCCACCTGCAGACGGACAGTGTTAAAGGCACCTTCACCCT
GCAGGACACTGGTGTTGGGATGAACAAAGAGGATCTGGTTTCTAACCTGGGCACGATTGCTCGATCTGGATCAAAGG
CTTTTCTGGACGCTCTGCAGAATCAAGCGGAGGCCAGCAGCTCCATCATTGGTCAGTTTGGTGTGGGCTTTTACTCC
GCCTTCATGGTGGCCGATAAGGTGGAGGTTTATTCTCAGTCGGCAGAAGCAGATGCTCCTGGATACAAGTGGTCCTC
AGACGGCTCTGGAGTGTTTGAGGTCGCTGAAGCTTCAGGTGTTCGACAGGGAACCAAGATTGTGCTCCACCTTAAAG
ACGACTGCAAAGAGTTTTCATCAGAGGACAGAGTTAAAGAGGTGGTGACCAAGTACAGCAACTTTGTGAGTTTCCCC
ATCTTCCTGAATGGACGGAGACTCAACACCCTGCAGGTACATGAAGTCTACATCTTGCCCAAGTGGCTGCGCTTCCT
ACGAGGTCAGAGAATCAAACCGTTTCAAACGCATGTTAGAAATCGTATCAGTCATGATAACATAAAATTACATCCAT
ATCTTCGGTTAGCTGAGATGATGAGCGAAGTGTTTACTAAAGACGATAATAATAAGGCGTGCCTGTTGTTTTGGTCT
CTGCAGGTCTTGTTCTGCTTCGAGCAGTTTGACGAGCTCACGCTTCTTCACCTCCGAGAGTTCGACAGGAAGAAGCT
GATCTCCGCTGAGACGGACATCGTTGTAGATCACTACAAAGAGGAGAAATTCCAGGACAGCAAGCCAGCATCTGAGC
GGTTGAGCAGTGAACAGGCTGAAGATCTGCTGGCCTGGATGAGGAACGCACTCGTTCAGAGAGTCACCAACATCAAG
GTGACTCCTCGTCTGGACACTCACCCGGCCATGATCACAGTGCTGGAGATGGGAGCCGCTCGACACTTTCTGCGCAC
ACAGCAGCTGGCCCGCAGCTCCGAGGAGAGAGCCCAGATCCTGCAGCCCACGCTGGAGATCAACACTGGGCATGATC
TGATCAAGAAGCTTCATGCGCTGAAGGACTCAAACCCTGAGCTGGCACAGCTTCTGCTCGAACAGATTTACGACAAC
GCCATGATCGCCGCTGGCCTGAACGAAGACCCTCGTCCGATGATCTCTCGACTGAATCAGCTGTTGACGCGAGCGCT
GGAGAAACACTGA
>(DRE)TRAP1P
MITAGGDTAPMEIHLQTDSVKGTFTLQDTGVGMNKEDLVSNLGTIARSGSKAFLDALQNQAEASSSIIGQFGVGFYS
AFMVADKVEVYSQSAEADAPGYKWSSDGSGVFEVAEASGVRQGTKIVLHLKDDCKEFSSEDRVKEVVTKYSNFVSFP
IFLNGRRLNTLQVHEVYILPKWLRFLRGQRIKPFQTHVRNRISHDNIKLHPYLRLAEMMSEVFTKDDNNKACLLFWS
LQVLFCFEQFDELTLLHLREFDRKKLISAETDIVVDHYKEEKFQDSKPASERLSSEQAEDLLAWMRNALVQRVTNIK
VTPRLDTHPAMITVLEMGAARHFLRTQQLARSSEERAQILQPTLEINTGHDLIKKLHALKDSNPELAQLLLEQIYDN
AMIAAGLNEDPRPMISRLNQLLTRALEKH
>(CIN)HSP90B1N
ATTATTAACTCACTGTACAGAAACAAAGAGATTTTCTTGAGAGAATTGATTTCTAATTCATCTGATGCGTTGGACAA
GATTCGATTACTTTCACTTACAGATGATGCAGCTCTTGCTGCCACAGATGAACTTTCCATTAAAGTTAAGGTTGACA
AAGAAAACAATGTTCTTCATGTTACGGACACTGGTATTGGAATGACAAAAGCTGACTTGATCAAAAATCTTGGAACC
ATCGCAAAATCTGGAACGAGTGATTTCTTCGAACAAATGTCGAAAGCATCTTCTGAAGATTCAGTGTCTGATCTCAT
TGGACAGTTTGGTGTTGGTTTCTACTCTGCATTCCTTATTGCCGACAAAGTTGTAGTGACATCCAAACACAACAACG
ACTCTCAACATATCTGGGAATCGGACTCTGAATCTTTCTCTGTTGTTGCCGATCCACGAGGTGACACTCTTGGCAGA
GGAACCACTGTCTCACTTTACTTGAAAGAAGAAGCATCAGACTATTTGGAAAATTCAACTGTGAAAGGACTGATTGA
AAAATATTCTCAATTCATTAACTTCCCCATCTACTTGTGGTCGGAGAAGACAATCAAAGAAGAAGTTCCTCTGGATG
AAGATGAAGCTGCAGAGGAGGAGAAACCTGAAGAAAAAGAAGCTTCGGATGAAGATGAGGAAGCCGAAGCAGAGGTG

GAAGAAGAAAAAGAAGACAAACCTAAAACAAAGACTGTAGAGAAGACTGTGTGGGATTGGGAACTTCTAAATGAAAT
GAAACCAGTGTGGCAGAGACCAGCTAAGGAAATCACTGACAAAGAATACAGTGATTTTTACAAATCTATCTCAAAAG
AAACCACTGACCCCATGGGTAAGACACACTTTGTAGCAGAAGGTGAAGTTTCGTTTAAATCGATTCTCTTCATCCCC
ATGACCTCCCCTGGTAACATGTTCCAAGATTATGGGCAGAAAAAGACAGATTTTATTAAGATGTACGTTCGTAGAGT
TTTCATCACCGATGACTTCCAAGACATGATGCCAAAATATTTGAGTTTCGTTCGTGGTGTGGTCGACAGTGACGATC
TTCCCCTTAATGTTTCGAGAGAAACTCTTCAACAACATAAACTGCTCAAAGTTATCAAGAAGAAGTTGGTGAGGAAA
GCACTCGATATGATCAAGAAGATCGATCCAGAGGTTTATCTCGAGAAATTCTGGAAAGAATTTGGAACAAACATCAA
ACTTGGTGTGATCGAAGATCATAGCAACCGTACAAGATTGGCAAAACTTCTTCGTTTCTTCACATCCAACCACCCAA
CTGATGTATCAAGTCTTGAACAATATGTTGAACGCATGAAGGAAAAACAGGAAAAGATTTATTTCTGTGCTGGCAAT
GGAAGAAAGGAGGTTGAAAATTCACCTTTTGTTGAGCGCTTATTGAAGAAGGGGTATGAAGTGATCTACCTCACAGA
GGCAGTGGATGAATACACCATTCAAGCTCTTCCTGAGTTTGATGGAAAGAGATTCCAAAATGTTGCCAAGGAAGGAC
TTGGGTTGGATGATGGGGAGAAGGCAAAGGAAAGAAAGGATGCTCTTGAGAAAGAATACGAACCACTTGCCAAGTGG
CTTAAAGAAACCGTGCTTACAGATAAGATTGAGAAGGCCGTTATCACTGAGCGCCTTACTGACTCACCATGTGCACT
TGTAGCCAGTCAATATGGATGGTCTGGCAACATGGAACGTATTATGAAAGCTCAGGCTTACCAAACACAAAAGGATT
CCACTAATAATTTCTATGCCAATCAGAAAAAGACACTTGAAATTAACCCACGTCATCCGCTGATCAAGAACTTGCTT
GAAAGAATAGAGACTGATGCTGAGGATGCACAAGCTGCACAGATCGCGAACGTGATGTTCGACACTGCTGCGCTTAG
ATCGGGATACTCCTTGAAAGATTCAGTTGATTTCTCAAAGAGAATTTTGGATATGTTGTACAAGAATCTTAACATTG
ATCCTGAAACTCCTATTGAGGAAGAACCTGAAGATGAAGAGCCAGAAGAGGAGGAAGAGGAAGAAGAGGTTGATACT
GATGATGAAGAGGAGGATGATGCAGAAGAGGCTGGTGGGGACGCAGATGAAACTACAGAAGAGCCGGAGCAAACCGA
AGCTGTGGAAGCTGAAAGTGAAGAGAGTCATGATGAATTA
>(CIN)HSP90B1P
IINSLYRNKEIFLRELISNSSDALDKIRLLSLTDDAALAATDELSIKVKVDKENNVLHVTDTGIGMTKADLIKNLGT
IAKSGTSDFFEQMSKASSEDSVSDLIGQFGVGFYSAFLIADKVVVTSKHNNDSQHIWESDSESFSVVADPRGDTLGR
GTTVSLYLKEEASDYLENSTVKGLIEKYSQFINFPIYLWSEKTIKEEVPLDEDEAAEEEKPEEKEASDEDEEAEAEV
EEEKEDKPKTKTVEKTVWDWELLNEMKPVWQRPAKEITDKEYSDFYKSISKETTDPMGKTHFVAEGEVSFKSILFIP
MTSPGNMFQDYGQKKTDFIKMYVRRVFITDDFQDMMPKYLSFVRGVVDSDDLPLNVSRETLQQHKLLKVIKKKLVRK
ALDMIKKIDPEVYLEKFWKEFGTNIKLGVIEDHSNRTRLAKLLRFFTSNHPTDVSSLEQYVERMKEKQEKIYFCAGN
GRKEVENSPFVERLLKKGYEVIYLTEAVDEYTIQALPEFDGKRFQNVAKEGLGLDDGEKAKERKDALEKEYEPLAKW
LKETVLTDKIEKAVITERLTDSPCALVASQYGWSGNMERIMKAQAYQTQKDSTNNFYANQKKTLEINPRHPLIKNLL
ERIETDAEDAQAAQIANVMFDTAALRSGYSLKDSVDFSKRILDMLYKNLNIDPETPIEEEPEDEEPEEEEEEEEVDT
DDEEEDDAEEAGGDADETTEEPEQTEAVEAESEESHDEL
>(CIN)HSP90B2N
ATGATTCGGAAGGTCTGGTTGTTCGTCTTCGTGGCAAGTTGTTTGTTGTTATCCGCCACACATGTTAAATCCGATGA
TGAGGGGACCACATCAGATGACACACCAAAAGTTGAACCAGATATTGGTAAAGCAGCTGATGGTTCAAAAACCGATG
ATGAAGTAGTTCAAAGGGAAGAAGAATCAATTCACTTGGATGGTTTAAGTCCAAGTGAAGTTAAACAACTTCGTGAA
CAATCAGAGAAACATGTTTTCCAAGCCGAGGTCAACCGAATGATGAAGCTGATTATTAACTCACTGTACAGAAACAA
AGAGATTTTCTTGAGAGAATTGATTTCTAATTCATCTGATGCGTTGGACAAGATCCGGTTACTTTCACTTACAGATG
ATGCAGCTCTTGCTGCCACAGATGAACTTTCCATTAAAGTTAAG
>(CIN)HSP90B2P
MIRKVWLFVFVASCLLLSATHVKSDDEGTTSDDTPKVEPDIGKAADGSKTDDEVVQREEESIHLDGLSPSEVKQLRE
QSEKHVFQAEVNRMMKLIINSLYRNKEIFLRELISNSSDALDKIRLLSLTDDAALAATDELSIKVK
>(CIN)TRAP1N
ATGGCCGCGCACACGAAAACGTGCTCCCAGGTTATTTTACGAAATATTTCTCGATTAACACCTAAAAGAATATCAAC
AAGAAGTGTATCTTATGCTGCTCAAACGCAAATACGCAGCGCTCAAAATGGTCATCTACGAAGAACCCACCTAAACA
AACTCCCAGCCAGTACTTGGCGGTTTTTATCAACGCAACCAGAGGCAACTAGTGAAGACACAGAAGAAGACTTGCAT
AATATAATCAAGGACGAAGAAAAAGTTGTTGGGCAAGCAAATGAACACGAGTTTCAAGCTGAAACAGCAGAATTGCT
GGATATTGTTGCTAAATCACTTTATTCCGAAAATGAGGTTTTTATCCGGGAAATTATCTCAAATGCGAGTGACGCGT
TAGAAAAACTTCGATATAATCGACTTACTACTGATGGTGAGGGTGGAGAAGCCCCACAAATGGAAATTCACATTCGC
ACCGATAAATATGAGAACACCTTCACAATCCAGGACACAGGTATTGGGATGACCAAGGAGGAATTATCTTCAAATCT
TGGAACTATTGCAAGATCAGGGTCAAAAGCGTTTCTCAAGCAAGTTGCAGATAAAGGAGATGCTGGTTCATCCATCA
TTGGACAGTTTGGTGTCGGTTTTTATTCAACCTTTATGGTTGGAAGTAAAGTTACTGTGTATACCAAATCTCATGAT
CCCAACAGCAAAGGGTACAGTTGGACCTCTGAAGGCGGTGTATCATATAAGATCACAGAAGCAGAATCTGTAATGCC
CGGAACCAAAATTGTGGTCACGCTGAAACCGGACTGCAGGAAATTTGCTGAAGAAGAGACCGTGAAGAGCATAATAA
AGAAACATAGTGCTTTTGTTGGATTTCCAGTAAGACTGAATGGAAAACTACTGAATGCCGTCAAGCCACTCTGGACA
CTTGACCCAAAGGACATTGACGAAGATCAACATTTAAATTTCTTTCGCCATCTGACAAACAACCAGAGTGACCATTA
TCTTTATAAATTGTTTTATAAGACGGATGCTCCATTAAATATAAGAAGTATTTTCTATGTATCTGAACAACAGCCTA
CCATGCTTGAAATGGCCAGGGATGCTTCTGGTATGTCGGGTGTATCATTGTACAGCAGGAAAGTTCTTGTCCAACAC

AAGACGACCAATCTCCTTCCAAAGTGGCTTCGCTTTTTAACAGGAGTAGTTGACAGTGAGGATATTCCGCTCAACTT
GAGCAGAGAACTGCTTCAAAACAGCGCTCTTATAAGTAAGCTGCGTGAGACACTGACAAGTCGTTTGATTCGTTACT
TTTTGGATCAAAGTCGTCGCGACCCTGAAAAATATTTAAAATTTCATGCCGGTTACAAACTGTTTATCACAGAAGGA
GTTTTGTCTGAAGACATGCAGGAAAAAAGGGAAGAAGTTGCTCAATTATTGAGATACGAATCATCCACTCTTCCTGA
AGGAGAAGTCACAAGTTTTAAAGATTACATTACAAGGATGGGAGAAGAACGTAACATTCTATATTTGTGTGCACCAA
GCCGAAGCTTAGCTGAATCTTCACCCTACTTTGAATCACTTAAAAACAGCGGAAGAGAAGTTTTGTTTTGCTATGAT
CCATATGATGAAGTTACTTTACTACAACTGAAAACCTACTCTGGGAAACAACTCTTCTCTCTTGAGAACGAAGTGGT
TGCCAATTCCTACCAAAATGAAAAGGACACAACACCAAAGGCAGATGGTGAATTGACAATGTCGGACTCACAGAGCA
AAGAATTGACTGATTGGGCAAAGGCAGCACTGGATCTGAAAGTCACAGATGTGAAGGTGACTGACAAACTTGACAAG
CATCCAGCAATGGTGACAGTATGGGAAATGGGTTCAGTTCGCCACTTCTTGAAAAGTCAGTATTTAACGGACCCCAA
AGGACTTTCTGAAAGTGAACGAACTGCTTTGTTTAAGCCAACACTTCAACTCAATGCTGTGCATCCAATTGTAATGA
AACTTACCTCTCTTAAGAACGAAGATGAAGATCTTGCCAAAGCTTTGCTGGAGCAGTTGTATGATAATGCTATGGTG
TCAGCTGGACTGTTTGAAGATGCTCGACCCATGGTCAATCGACTCAATGACCTGCTTACTAAAGTACTTCAGAAGCA
CTAA
>(CIN)TRAP1P
MAAHTKTCSQVILRNISRLTPKRISTRSVSYAAQTQIRSAQNGHLRRTHLNKLPASTWRFLSTQPEATSEDTEEDLH
NIIKDEEKVVGQANEHEFQAETAELLDIVAKSLYSENEVFIREIISNASDALEKLRYNRLTTDGEGGEAPQMEIHIR
TDKYENTFTIQDTGIGMTKEELSSNLGTIARSGSKAFLKQVADKGDAGSSIIGQFGVGFYSTFMVGSKVTVYTKSHD
PNSKGYSWTSEGGVSYKITEAESVMPGTKIVVTLKPDCRKFAEEETVKSIIKKHSAFVGFPVRLNGKLLNAVKPLWT
LDPKDIDEDQHLNFFRHLTNNQSDHYLYKLFYKTDAPLNIRSIFYVSEQQPTMLEMARDASGMSGVSLYSRKVLVQH
KTTNLLPKWLRFLTGVVDSEDIPLNLSRELLQNSALISKLRETLTSRLIRYFLDQSRRDPEKYLKFHAGYKLFITEG
VLSEDMQEKREEVAQLLRYESSTLPEGEVTSFKDYITRMGEERNILYLCAPSRSLAESSPYFESLKNSGREVLFCYD
PYDEVTLLQLKTYSGKQLFSLENEVVANSYQNEKDTTPKADGELTMSDSQSKELTDWAKAALDLKVTDVKVTDKLDK
HPAMVTVWEMGSVRHFLKSQYLTDPKGLSESERTALFKPTLQLNAVHPIVMKLTSLKNEDEDLAKALLEQLYDNAMV
SAGLFEDARPMVNRLNDLLTKVLQKH
